# DNA methylation age in paired tumor and adjacent normal breast tissue in Chinese women with breast cancer

**DOI:** 10.1186/s13148-023-01465-1

**Published:** 2023-03-30

**Authors:** Hela Koka, Clara Bodelon, Steve Horvath, Priscilla Ming Yi Lee, Difei Wang, Lei Song, Tongwu Zhang, Amber N. Hurson, Jennifer Lyn Guida, Bin Zhu, Maeve Bailey-Whyte, Feng Wang, Cherry Wu, Koon Ho Tsang, Yee-Kei Tsoi, W. C. Chan, Sze Hong Law, Ray Ka Wai Hung, Gary M. Tse, Karen Ka-wan Yuen, Eric Karlins, Kristine Jones, Aurelie Vogt, Bin Zhu, Amy Hutchinson, Belynda Hicks, Montserrat Garcia-Closas, Stephen Chanock, Jill Barnholtz-Sloan, Lap Ah Tse, Xiaohong R. Yang

**Affiliations:** 1 Division of Cancer Epidemiology and Genetics, National Cancer Institute, NIH, DHHS, Bethesda, MD USA; 2 Department of Human Genetics, David Geffen School of Medicine, University of California Los Angeles, Los Angeles, CA USA; 3 San Diego Institute of Science, Alto Labs, San Diego, CA USA; 4 The Jockey Club School of Public Health and Primary Care, The Chinese University of Hong Kong., Prince of Wales Hospital, Sha Tin, N.T., Hong Kong SAR China; 5 Cancer Genomics Research Laboratory, Leidos Biomedical Research, Frederick National Laboratory for Cancer Research, Frederick, MD USA; 6 Division of Cancer Control and Population Sciences, National Cancer Institute, NIH, DHHS, Bethesda, MD USA; 7 School of Medicine, University of Limerick, Limerick, Ireland; 8 Department of Pathology, North District Hospital, Hong Kong, China; 9 Department of Pathology, Yan Chai Hospital, Hong Kong, China; 10 Department of Surgery, North District Hospital, Hong Kong, China; 11 Department of Anatomical and Cellular Pathology, The Chinese University of Hong Kong, Hong Kong, China; 12 Center for Biomedical Informatics and Information Technology, National Cancer Institute, NIH, DHHS, Bethesda, MD USA

**Keywords:** Asian, Breast cancer subtype, DNA methylation, Epigenetic aging, Genomic characteristics

## Abstract

**Background:**

Few studies have examined epigenetic age acceleration (AA), the difference between DNA methylation (DNAm) predicted age and chronological age, in relation to somatic genomic features in paired cancer and normal tissue, with less work done in non-European populations. In this study, we aimed to examine DNAm age and its associations with breast cancer risk factors, subtypes, somatic genomic profiles including mutation and copy number alterations and other aging markers in breast tissue of Chinese breast cancer (BC) patients from Hong Kong.

**Methods:**

We performed genome-wide DNA methylation profiling of 196 tumor and 188 paired adjacent normal tissue collected from Chinese BC patients in Hong Kong (HKBC) using Illumina MethylationEPIC array. The DNAm age was calculated using Horvath’s pan-tissue clock model. Somatic genomic features were based on data from RNA sequencing (RNASeq), whole-exome sequencing (WES), and whole-genome sequencing (WGS). Pearson’s correlation (*r*), Kruskal–Wallis test, and regression models were used to estimate associations of DNAm AA with somatic features and breast cancer risk factors.

**Results:**

DNAm age showed a stronger correlation with chronological age in normal (Pearson *r* = 0.78, *P* < 2.2e−16) than in tumor tissue (Pearson *r* = 0.31, *P* = 7.8e−06). Although overall DNAm age or AA did not vary significantly by tissue within the same individual, luminal A tumors exhibited increased DNAm AA (*P* = 0.004) while HER2-enriched/basal-like tumors exhibited markedly lower DNAm AA (*P* = < .0001) compared with paired normal tissue. Consistent with the subtype association, tumor DNAm AA was positively correlated with *ESR1* (Pearson *r* = 0.39, *P* = 6.3e−06) and *PGR* (Pearson *r* = 0.36, *P* = 2.4e−05) gene expression. In line with this, we found that increasing DNAm AA was associated with higher body mass index (*P* = 0.039) and earlier age at menarche (*P* = 0.035), factors that are related to cumulative exposure to estrogen. In contrast, variables indicating extensive genomic instability, such as *TP53* somatic mutations, high tumor mutation/copy number alteration burden, and homologous repair deficiency were associated with lower DNAm AA.

**Conclusions:**

Our findings provide additional insights into the complexity of breast tissue aging that is associated with the interaction of hormonal, genomic, and epigenetic mechanisms in an East Asian population.

**Supplementary Information:**

The online version contains supplementary material available at 10.1186/s13148-023-01465-1.

## Background

Globally, breast cancer is the leading cause of cancer incidence and deaths among females [1]. Chronological age is a well-established risk factor for breast cancer. However, the chronological age of an individual may not reflect the true biological age of a specific organ, such as the breast. Indeed, individuals of the same chronological age may undergo biological processes at different rates [2]. Epigenetic markers, such as DNA methylation (DNAm), in a specific organ may capture the cumulative effects of endogenous and exogenous exposures in that organ and therefore may be a better proxy for the age of the organ than chronological age.

Several epigenetic age estimators have been developed using DNA methylation levels in a few loci in the human genome, and these estimators are highly correlated with chronological age. A recent study investigating hormonal factors and these epigenetic age estimators in healthy breast tissues reported that earlier age at menarche and higher body mass index (BMI) were associated with increased DNAm age acceleration (AA), defined as a positive deviation of epigenetic age from chronological age [3]. These findings suggest that exposure to cumulative estrogen may accelerate DNAm age. Several studies have looked at DNAm AA and breast cancer subtypes, which are also related to cumulative estrogen. In a recently published work, Castle et al. developed a breast tissue-specific epigenetic clock using next-generation sequencing data and found that DNAm age acceleration was seen in hormone receptor positive and human epidermal growth factor receptor-2+ (HER2+) breast cancer subtypes but not in triple negative breast cancers (TNBC) [4]. These results agree with the findings using a pan-cancer tissue DNAm measure (Horvath clock) in The Cancer Genome Atlas (TCGA) data, which showed that DNAm AA was positively associated with hormone receptor positivity but negatively associated with tumor mutational burden (TMB) and *TP53* mutations in breast tumors [5]. However, TCGA does not represent the general patient population, particularly non-European subjects. Previous studies have shown that breast cancer in Asian women may manifest differently compared to their Western counterparts with respect to the earlier age at onset and higher prevalence of luminal B and HER2-positive breast cancer subtypes [6], suggesting that breast tissue aging in Asian women may exhibit different patterns from those observed in European women. Investigating epigenetic age in normal breast tissue and paired tumors and its association with patient and tumor characteristics in diverse populations may improve our understanding of genomic and epigenomic processes that drive the complex tissue aging in breast cancer and its subtypes. To that end, we estimated DNAm age in breast tumor and paired adjacent normal tissues of an East Asian population and related DNAm AA with cancer genomic features and breast cancer risk factors.

## Materials and methods

### Hong Kong breast cancer (HKBC) study participants

The information on the study design and biospecimen collection included in this work has been previously described [7]. Briefly, breast tumor and paired tumor-adjacent normal fresh frozen breast tissue samples were collected from treatment-naïve breast cancer patients diagnosed and treated in two Hong Kong (HK) hospitals between 2013 and 2016. Participants were included based on the criteria: (a) being female, (b) between 20 and 84 years old, (c) diagnosed with breast cancer no more than 3 months prior to the recruitment interview and histologically confirmed (International Classification of Disease, Tenth Revision, code 50), and (d) of Chinese ethnicity and resident of Hong Kong for at least 5 years. Patients with pre-surgery treatment were excluded from the study. Medical records and questionnaires were utilized to retrieve clinical characteristics and breast cancer risk factors for each patient. The current study included 196 tumor and 188 paired normal with methylation profiling data available. The study protocol was approved by the ethics committees of the Joint Chinese University of Hong Kong-New Territories East Cluster, the Kowloon West Cluster, and the National Cancer Institute (NCI). All subjects provided a written informed consent prior to the surgery.

### DNA methylation analysis and DNA methylation age estimation

Pathology review and DNA extraction were conducted at the Biospecimen Core Resource (BCR, Nationwide Children’s Hospital, Columbus, OH) on paired tumor and adjacent normal breast tissues samples. DNA was extracted only on tumor samples with > 50% tumor cells and on normal tissue with 0% tumor cells. Methylation profiling was performed using the Infinium MethylationEPIC BeadChip (Illumina, San Diego, CA) at the Cancer Genomics Research Laboratory (CGR). Quality control (QC) was performed using the basic intensity R package minfi. Raw methylated and unmethylated intensities were background corrected and dye-bias-equalized to correct for technical variation in signal between arrays.

To calculate DNA methylation age, we employed a pan-tissue clock, a model proposed by Horvath (https://dnamage.genetics.ucla.edu) [5] since it applies to female breast tissue [3, 8]. Horvath clock combines information from 353 CpGs, and it was designed using DNA methylation data from multiple tissue types, which makes this model robust for the breast tissue samples.

### Additional genomic data

**Fig. 1:**
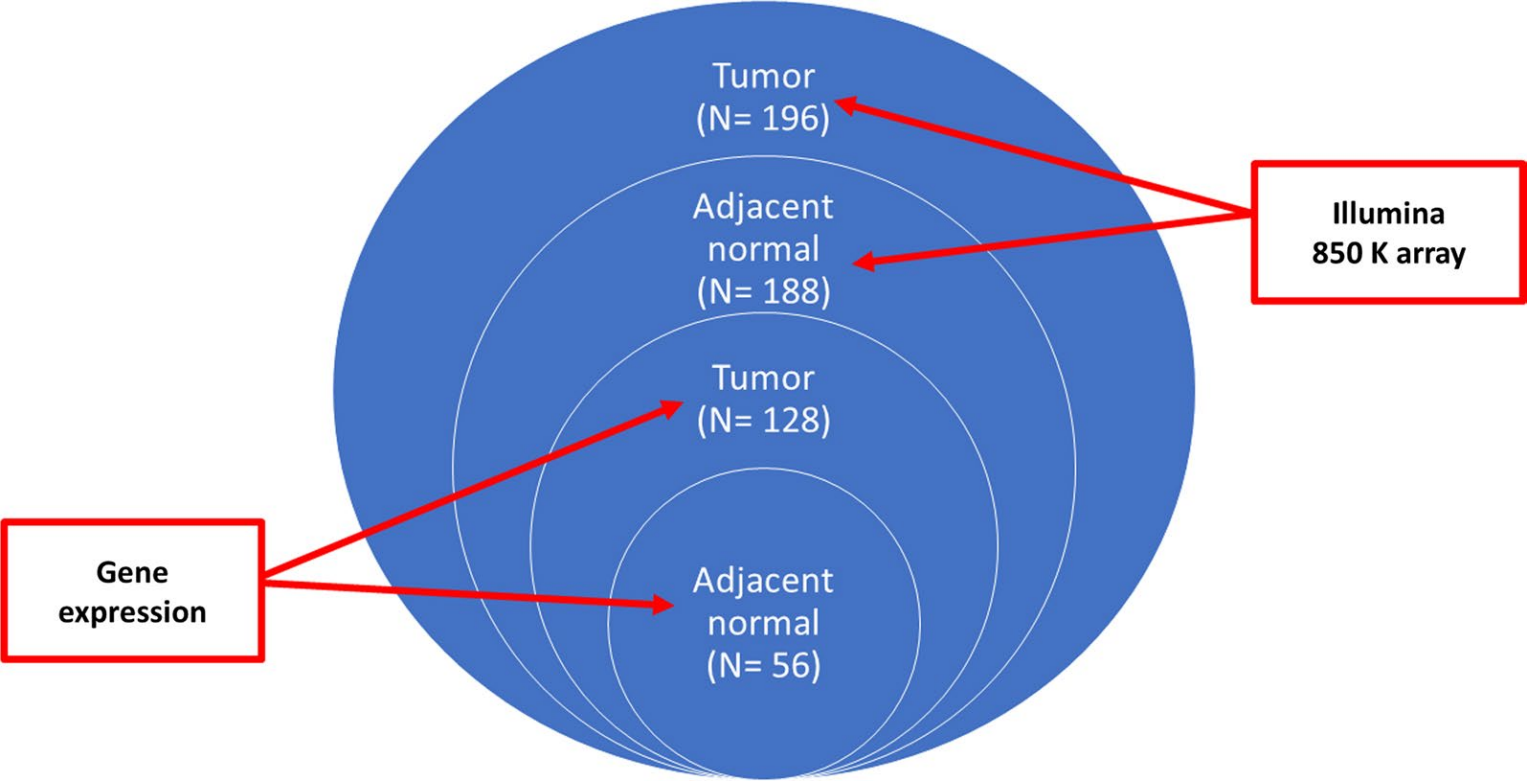
Sample size and data type in the Hong Kong breast cancer (HKBC) study

RNA sequencing (RNASeq), whole-exome sequencing (WES), and whole-genome sequencing (WGS) data were available for most of these patients (Fig. 1). Sequencing methods and bioinformatic analyses were described previously [9–11].

PAM50 subtype (luminal A, luminal B, HER2-enriched, basal-like, and normal-like) was defined by an absolute intrinsic subtyping (AIMS) method using RNASeq data [12]. Normal-like tumors were not included in the stratified analyses due to inadequate sample size, but they were analyzed in overall analyses. For patients without RNASeq data (*n* = 6), subtype was defined using the immunohistochemical status of estrogen receptor (ER), progesterone receptor (PR), and HER2. Breast cancer subtypes using immunohistochemical data were defined as follows: luminal A-like (ER+/PR+/HER2−); luminal B-like (ER+/PR−; or ER−/PR+; and/or HER2+); HER2-enriched (ER−/PR−/HER2+); and TNBC (ER−/PR−/HER2−). Among six patients, whose tumor subtype was defined immunohistochemically, four of them were of luminal A, one of them was of HER2-enriched type, and one was defined as TNBC subtype (we grouped the TNBC tumor with basal-like ones in the analysis).

Methods for somatic mutation and copy number alteration (SCNA) analyses, based on WES/WGS data, were previously described in our previous HKBC genomic study [10]. WGS-based analyses such as mutational signature, telomere length, and homologous recombination deficiency (HRD) were described in detail in Zhang et al. [11]. We measured HRD and the percent genome influenced by somatic copy number alterations (PGS) as markers for genomic instability. HRD, which leads to accumulation of genomic aberrations that manifest as genomic instability, is associated with BRCAness and is a molecular biomarker for administrating PARP inhibitor (PARPi) or platinum-based (Pt) chemotherapy in breast cancer [13]. HRDetect score was estimated by combining single nucleotide variation (SNV) signature 3, SNV signature 8, structural variant (SV) signature 3, SV signature 5, HRD index from copy number profile, and deletion fractions with microhomology, all associated with HRD [14]. HRDetect score was dichotomized using a pre-determined cutoff point of 0.7 [14]. In addition to classifying these tumors being mutant (Mut) or wildtype (WT) for a *TP53* mutation using DNA sequencing data, we also classified them as mutant-like (Mut-like) or wildtype-like (WT-like) using a validated RNA-based method which combines information from *TP53*-dependent genes and reflects *TP53* pathway activity [15]. The expression-based p53 loss-identifying signature is shared by different BC subtypes and provides prognostic information [15].

Other aging-related makers included the two COSMIC clock-related mutational signatures based on single-base substitution (SBS), SBS1 and SBS5, and telomere length, both estimated using WGS. In addition, we also evaluated the expression of *CDKN2A*, a marker of cellular senescence, from RNASeq data.

Tumor purity was assessed using the ESTIMATE algorithm using RNASeq data [16] to adjust the analyses of tumor genomic features.

### Statistical analysis

DNAm AA was estimated to be the residual from regressing DNA methylation age on chronological age, for each tissue type. Pearson correlation was used to estimate correlations between DNAm age or DNAm AA and different characteristics such as chronological age, gene expression levels, proportions of mutational signatures, and telomere length. Kruskal–Wallis test was performed to evaluate differences of median DNAm AA across different groups. Logistic regression models were used to assess the associations between risk factors/somatic genomic features and DNAm AA in tumor and normal tissue, respectively, where DNAm AA was the independent variable and adjusted for tumor stage and purity (for tumor samples) and percent area of fat on tissue slide (for normal breast tissue samples). Generalized linear regression modeling was used to evaluate the impact of tumor characteristics on tumor tissue DNAm AA. To determine which of the variables among molecular subtype, *TP53* mutation status, tumor mutational burden (TMB), *ESR1* (estrogen receptor 1) expression, and PGS was predictive of DNAm AA when accounting for each other, we modeled DNAm AA as the outcome variable and included the genomic factors jointly as explanatory variables. All statistical tests were two-sided and performed using SAS version 9.4 (SAS Institute, Cary, NC, USA) or R version 3.6.3 (R Foundation for Statistical Computing, Vienna, Austria).

## Results

### Study populations

Our study population was comprised of 196 breast cancer patients from the Hong Kong breast cancer study (HKBC). (Fig. 1 and Table 1). Most patients were older than 50 years old and had early-stage (I or II) and luminal tumors (Table 1).

**Table 1:** Patient characteristics in Hong Kong (HKBC) dataset

	HKBC (N = 196)
Characteristic	N (%)
Age, year	
< 50	37 (18.9)
50–60	58 (29.5)
≥ 60	101 (51.6)
BMI^a^, kg/m^2^	
< 25	95 (55.2)
25–30	55 (32.0)
≥ 30	22 (12.8)
Age at menarche, year	
< 14	100 (54.1)
≥ 14	85 (45.9)
Parity	
Nulliparous	17 (9.1)
Parous	169 (90.9)
Age at first birth, year^b^	
< 25	61 (38.6)
≥ 25	97 (61.4)
Menopausal status	
Pre	43 (23.2)
Post	142 (76.8)
Age at menopause, year^c^	
≤ 50	63 (53.4)
> 50	55 (46.6)
PAM50^d^	
Luminal A	70 (41.2)
Luminal B	51 (30.0)
HER2-enriched	28 (16.5)
Basal	21 (12.3)
Clinical stage	
I/II	91 (85.1)
III/IV	16 (14.9)
TP53 mutation status	
No	124 (72.1)
Yes	48 (27.9)

^a^*BMI* Body mass index

^b^Among parous women

^c^Among menopausal women

^d^AIMS was used to infer tumor subtype

### DNAm age and its acceleration

**Fig. 2:**
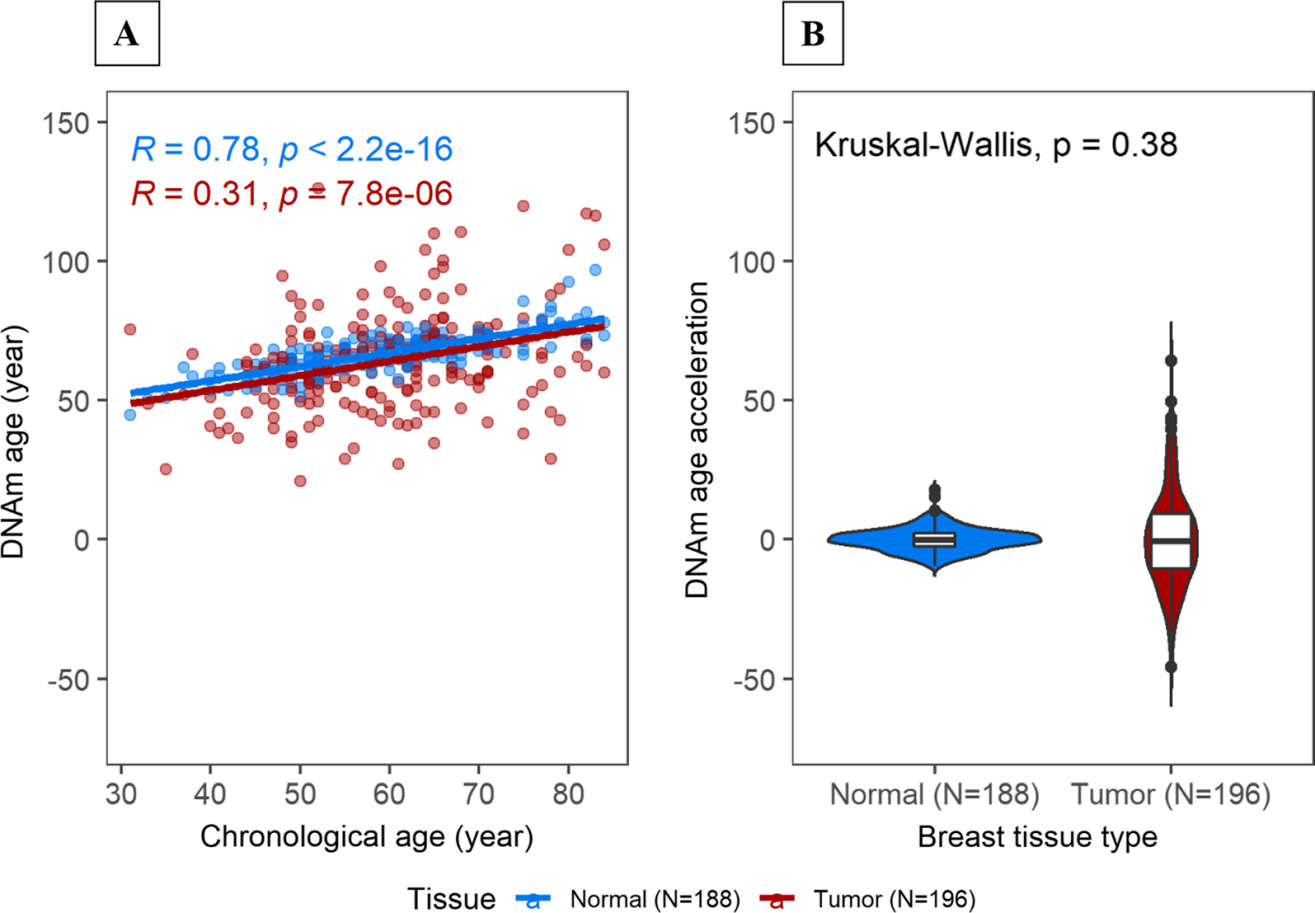
DNAm age and DNAm age acceleration in tumor and adjacent normal breast tissue. **A** Correlation of DNAm age with chronological for each tissue type (blue = normal, red = tumor); **B** Distribution of DNAm age acceleration by tissue type. Pearson correlation coefficient (*R*) and *P* values were computed for each tissue type. Kruskal–Wallis test was used to formally assess median differences in DNAm age acceleration across tissue samples

Consistent with previous data, Horvath’s estimated DNAm age was highly correlated with chronological age in normal breast tissue samples (Pearson *r* = 0.78, *P* < 2.2e−16; Fig. 2A). In tumor samples, as expected, DNAm age showed a weaker correlation with chronological age (Fig. 2A). We observed no significant age acceleration in either tumor or adjacent normal tissue (Fig. 2B).

### DNAm age acceleration and breast cancer subtypes

**Fig. 3:**
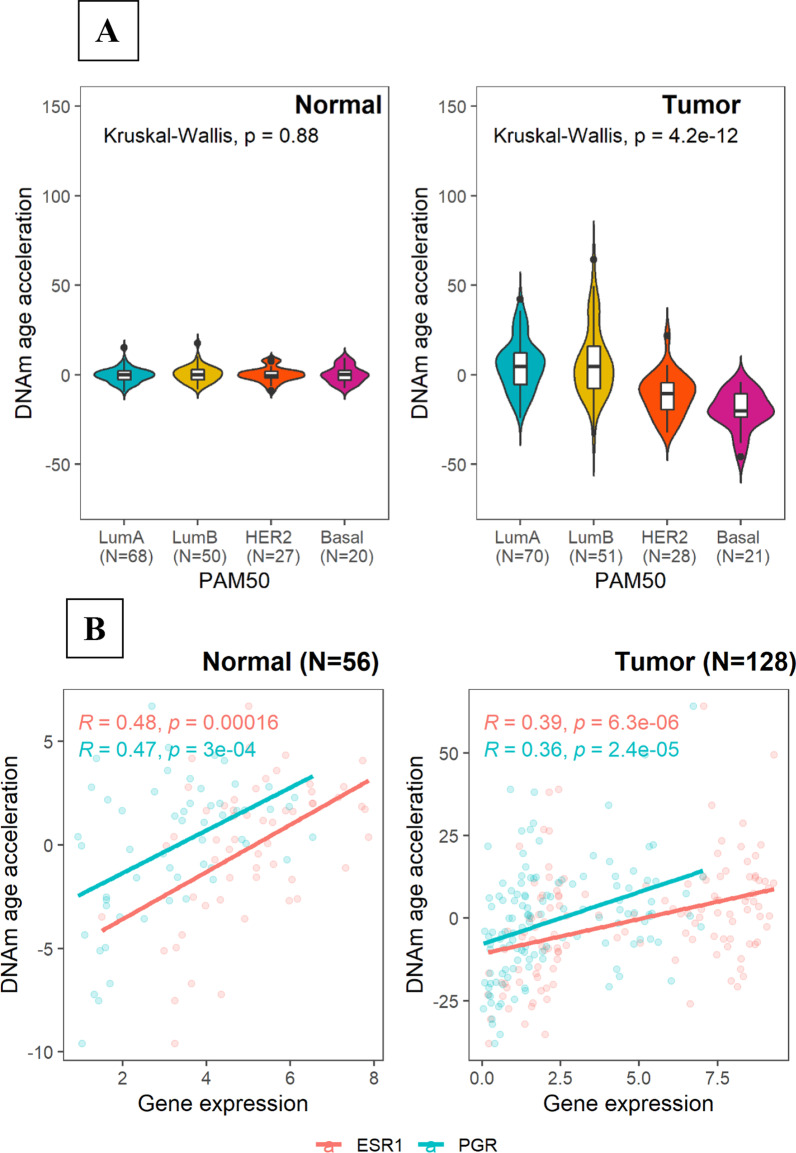
Relation between DNAm age acceleration and breast cancer subtypes. **A** Distribution of DNAm age acceleration in normal and tumor tissue by breast cancer subtypes; **B** Associations between DNAm age acceleration and *ESR1* and *PGR* gene expression in normal and tumor tissue. Kruskal–Wallis test was used to formally assess median differences by tumor subtype. Pearson correlation test was utilized to test for significance of the correlation between gene expression and DNAm age acceleration

Overall, the median DNAm AA was not significantly different between tumor and histological normal tissue (Fig. 2B); however, luminal A tumors exhibited increased DNAm AA (*P* = 0.004) while HER2-enriched/basal-like tumors exhibited markedly lower DNAm AA (*P* = < 0.0001) compared with paired normal tissue. In addition, DNAm AA in tumors showed greater variations than that in normal tissue and significantly differed by breast cancer subtype. Specifically, HER2-enriched or basal-like tumors were more likely to have lower median DNAm AA compared to that in luminal breast tumors (Kruskal–Wallis *P* = 4.2e−12; Fig. 3A). Subtype differences in DNAm AA remained statistically significant when we adjusted for tumor purity and stage. In adjacent normal tissue, DNAm AA did not vary by tumor subtype (Kruskal–Wallis *P* = 0.88; Fig. 3A).

Given the observed difference with breast cancer tumor subtypes, we investigated DNAm AA with respect to gene expression levels of *ESR1* (estrogen receptor 1) and *PGR* (progesterone receptor) in these women. We found that DNAm AA was positively associated with *ESR1* and *PGR* gene expression in both tumor and normal tissue samples (Fig. 3B), suggesting that the activation of estrogen signaling pathway may accelerate breast tissue aging.

### DNAm age acceleration and genomic features

**Fig. 4:**
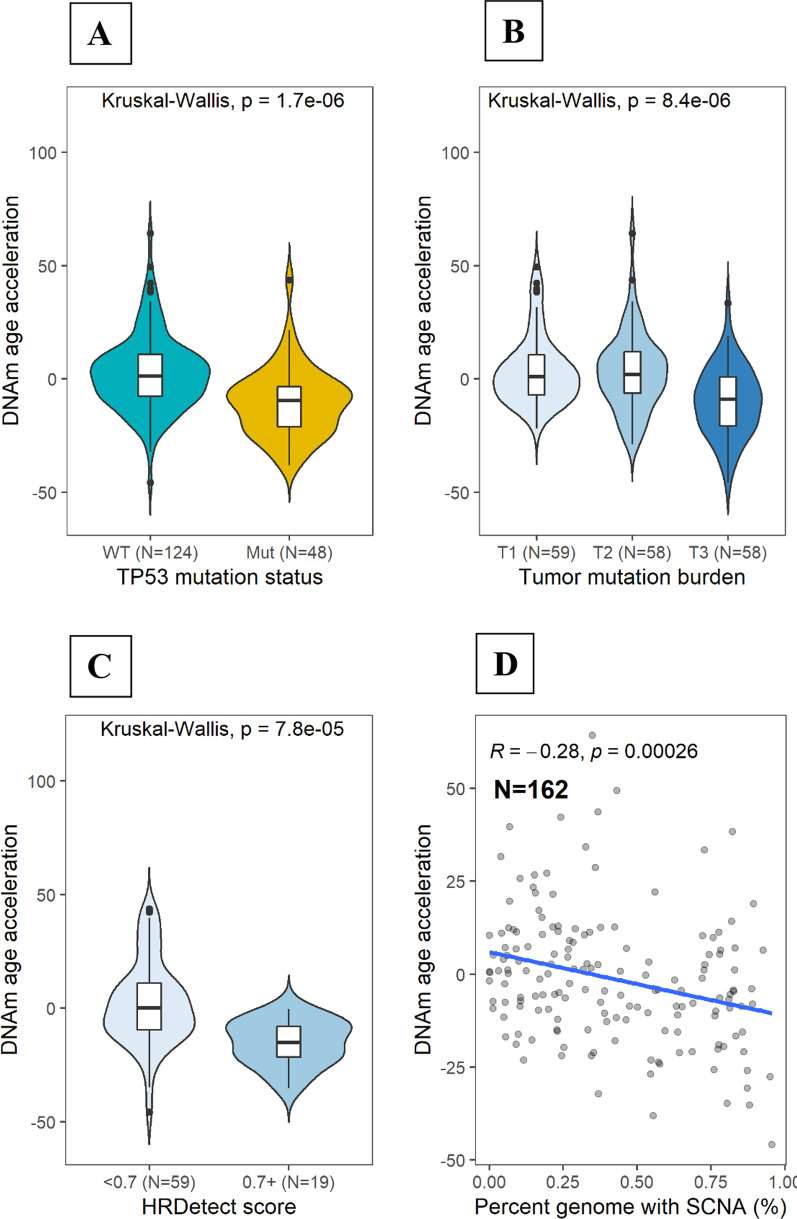
Distributions of DNAm age acceleration by genomic features. **A** Distribution of DNAm age acceleration by DNA-based *TP53* mutation status. **B** Distribution of DNAm age acceleration by tumor mutation burden categorized using tertials (*T*1 = < 1.0, *T*2 = 1:0–1.95, *T*3 = ≥ 1.95); **C** Distribution of DNAm age acceleration by HRDetect score (dichotomized using 70% as a cutoff); **D** Distribution of DNAm age acceleration by percent genome with somatic copy number of alterations (SCNAs). Kruskal–Wallis test was used to formally assess median differences by each genomic feature, separately. Pearson correlation test was utilized to test for significance of linear relationship between percent genome with SCNAs and DNAm age acceleration

Taking advantage of the DNA (WGS or WES) and RNA sequencing data we have for the cancer patients in this study, we looked at several genomic features in relation to epigenetic aging. We found that DNAm AA in tumor tissue was negatively correlated with measures of tumor aggressiveness and genomic instability, including the presence of *TP53* mutations (Fig. 4A), mutant-like TP53 functional status based on gene expression data (see Methods, Additional file 1: Figure S1) [15], higher tumor mutational burden (TMB, Fig. 4B), higher HRDetect score (a mutational signature-based score to predict homologous repair deficiency) (Fig. 4C), and increased percent genome influenced by somatic copy number alterations (PGS) (Fig. 4D). Given that HER2-enriched and basal-like tumors are more likely to have higher TMB, *TP53* mutations, and genomic instability, it is possible that the associations between these genomic features and DNAm AA might be mediated through molecular subtypes. To address this question, we modeled DNAm AA as the outcome variable and molecular subtype, *ESR1* expression, *TP53* mutation status, TMB, and PGS jointly as explanatory variables, and we found that *TP53* and TMB showed the most significant associations with DNAm AA (Table 2). When examining these associations separately in each subtype, we found that none of the examined genomic features was associated with DNAm AA within luminal A tumors, while *TP53* mutation status and TMB showed correlation with DNAm AA among other subtypes (Additional file 2: Figure S2, Additional file 5: Table S1). Similar results were observed in breast tumors from TCGA (*n* = 559, Additional file 3: Figure S3, Additional file 6: Table S2). These results suggest that *TP53* mutations may influence epigenetic aging independently of molecular subtype. While hormone receptor expression and *TP53* mutations are likely the driving force of the observed different direction of DNAm AA between luminal A and basal-like tumors, additional mechanisms may exist that contribute to variations of DNAm AA within luminal A tumors.

**Table 2:** Associations between DNAm age acceleration in tumor tissue and tumor characteristics in Hong Kong breast cancer women

Characteristic	*β* (SE)^a^	*P* value^a^
Tumor subtype		
Luminal A	Ref.	−
Luminal B	5.11 (3.1)	0.098
HER2	− 7.92 (4.2)	0.064
Basal	− 9.87 (5.2)	0.059
*TP53*- Mut vs. *TP53*- WT	− 9.89 (3.5)	**0.006**
Tumor mutation burden	− 0.58 (0.2)	**0.017**
PGS^b^	− 6.29 (4.8)	0.195
*ESR1*	0.81 (0.5)	0.084

Bold denotes a statistical significant result with* P*-value < 0.05

^a^Results were obtained from running multivariate linear regression analysis, where age acceleration was treated as the outcome

^b^Percent genome with copy number alterations

**Fig. 5:**
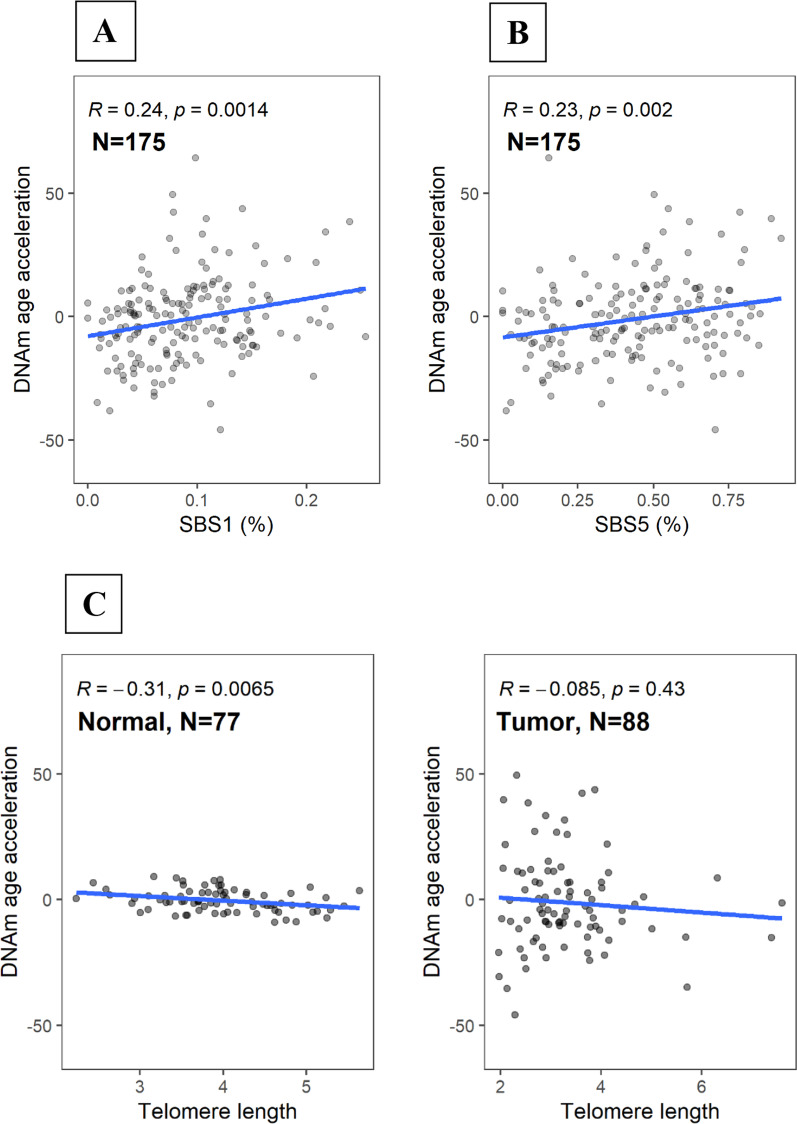

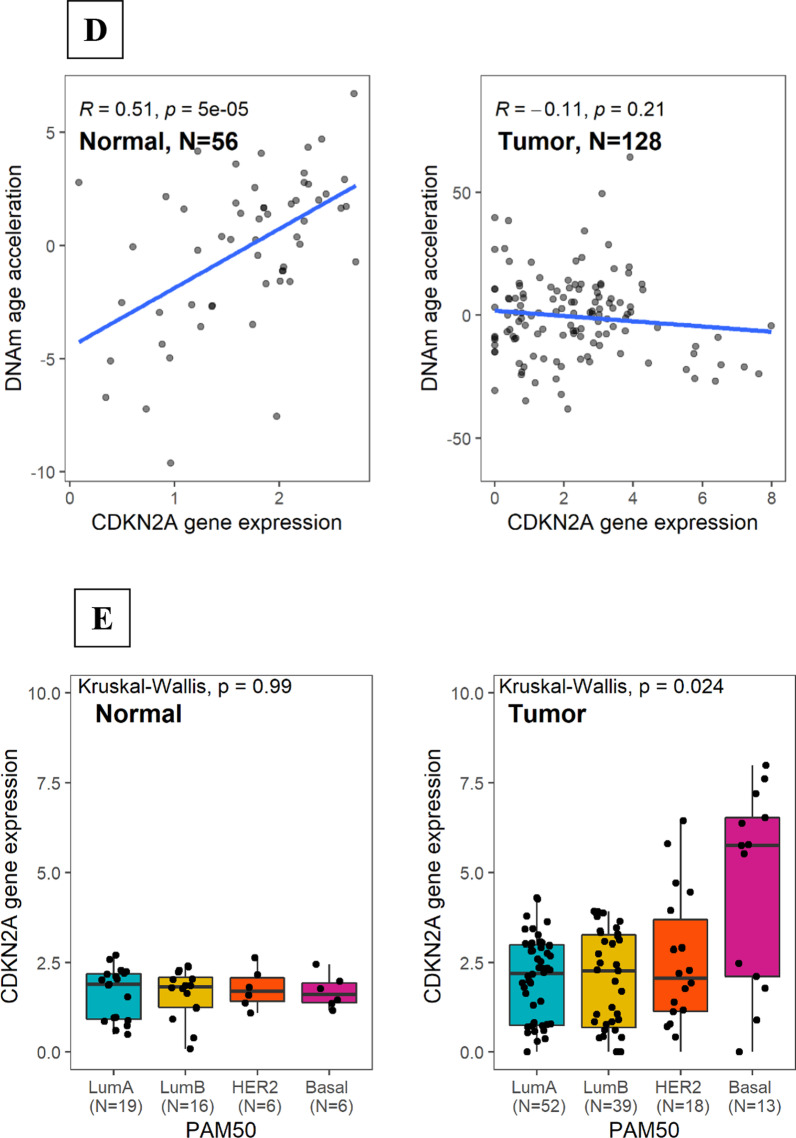
DNAm age acceleration and aging markers in normal and tumor tissue. Correlations between DNAm age acceleration in tumor tissue and single-base substitution (SBS) COSMIC mutational signatures **A** SBS 1; **B** SBS 5; **C** Association between DNAm age acceleration and telomere length (estimated using WGS data) stratified by tissue type; **D** Association between DNAm age acceleration and *CDKN2A* gene expression; **E** Distribution of *CDKN2A* gene expression in normal and tumor tissue. Pearson correlation test was utilized to test for significance of the correlation between each marker and DNAm age acceleration, separately

Overall, we observed weak but consistent correlations between DNAm AA and other aging markers. Notably, DNAm AA in tumor tissue was positively correlated with two clock-like COSMIC mutational signatures SBS1 and SBS5 **(**Fig. 5A, B). There was a negative correlation between telomere length and DNAm AA in normal (Pearson *r*_Normal_ = − 0.31, *P* = 0.007) but not in tumor tissue (Pearson *r*_Tumor_ = − 0.09, *P* = 0.43; Fig. 5C). We also found positive correlations between DNAm AA and the expression of *CDKN2A*, a marker of cellular senescence (Pearson *r*_Normal_ = 0.51, *P* = 5e−05, Fig. 5D), which again, were only seen in normal tissue. In tumor tissue, the associations varied by tumor subtype, with basal-like tumors showing negative correlations with DNAm AA (Additional file 2: Figure S2). Interestingly, we observed significantly higher *CDKN2A* expression levels in basal-like tumors as compared to tumors of other subtypes (Fig. 5E). The upregulation of *CDKN2A* and its negative correlation with DNAm AA may suggest extensive cellular senescence in basal-like tumors that may lead to dysregulations of epigenomic processes.

To address whether DNAm age estimation was influenced by SCNAs in the CpG sites used in Horvath’s clock, which might have led to the inaccurate measurement of methylation levels at these loci, we excluded tumors displaying extensive SCNAs in the Horvath clock sites in a sensitivity analysis. Overall, the results did not differ significantly from those based on all subjects (Additional file 4: Figure S4).

### DNAm age acceleration and breast cancer risk factors

**Fig. 6:**
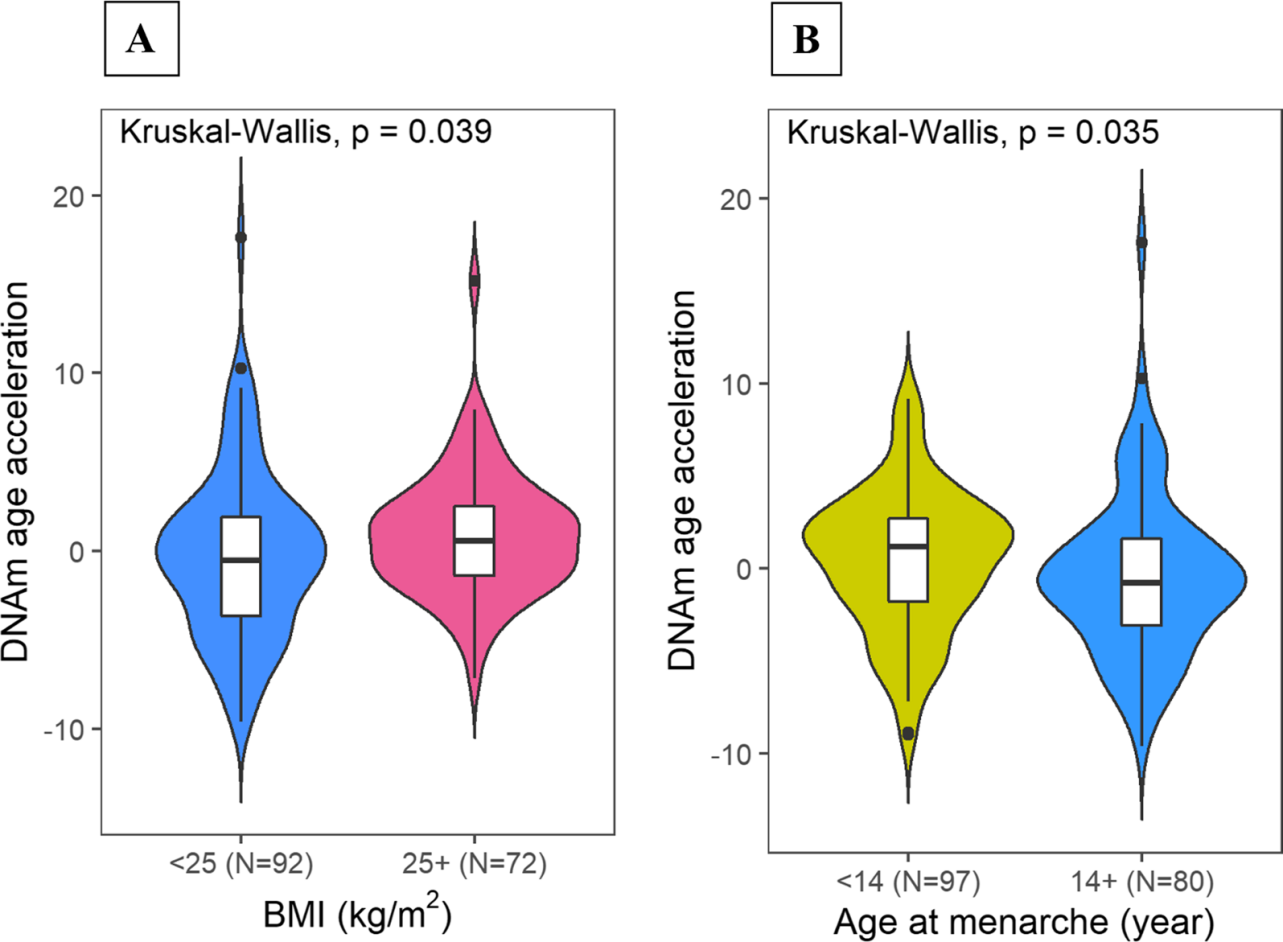
DNAm age acceleration and established breast cancer risk factors. Associations between DNAm age acceleration in normal tissue and **A** body mass index (BMI) and **B** age at menarche. Kruskal–Wallis test was used to formally assess median differences in different groups categorizing each risk factor

Finally, we investigated normal tissue DNAm AA in relation to several established breast cancer risk factors such as reproductive characteristics and BMI. Consistent with previous findings, we found that DNAm AA was associated with higher BMI and earlier age at menarche (Fig. 6, Additional file 7: Table S3). In sensitivity analyses, we further adjusted for percent fat area on tissue slide and found the association with BMI remained significant (*P* = 0.023), while the association for age at menarche became nonsignificant (*P* = 0.409).

## Discussion

In this study, using a pan-tissue DNAm age estimator and leveraging extensive genomic data from breast cancer patients in an East Asian population, we estimated epigenetic aging in human breast tissue from tumor as well as cancer adjacent normal breast tissue samples, and investigated whether the DNA methylation-based age acceleration of the diseased breast tissue differed by breast cancer subtype and other genomic characteristics. Consistent with findings from a previous analysis primarily based on European samples [5], DNAm AA showed a different direction in luminal and non-luminal tumors, with an age acceleration in luminal A and luminal B tumors and an age deceleration in HER2-enriched and basal-like tumors. In line with this, we found that DNAm AA was positively associated with *ESR1* and *PGR* expression but negatively associated with tumor aggressiveness and genomic instability measures, including *TP53* mutation status/RNA-based functional status, tumor mutational burden, HRD score, and percent of genome influenced by SCNAs. Our data corroborated previously reported associations between DNAm age and breast cancer subtypes, TMB, and *TP53* mutations, which were primarily based on TCGA data [5], in our East Asian population and extended the investigation by comprehensively analyzing DNAm AA in relation to additional genomic alterations, other aging markers, and breast cancer etiologic factors.

A recently published work by Castle et al. reported a higher DNAm AA in breast tumor compared to the adjacent normal and normal breast tissue [4]. Using Horvath clock, we did not observe significant DNAm age acceleration in cancer patients’ tumor or adjacent normal tissue. However, there was a weaker correlation between epigenetic and chronological age in in tumor than in normal tissue. This is not surprising since molecular processes associated with normal cellular functions and aging, such as proliferation, apoptosis, and inflammation are dysregulated in cancer cells. Therefore, the DNA methylation pattern that underpins epigenetic clocks is likely to be different and more heterogeneous in tumor than that of normal cells, leading to disrupted age estimates. Consistently, we observed weak but expected correlations between DNAm AA and other aging markers such as shortened telomere length and increased cell senescence in normal but not in tumor tissue.

Interestingly, when stratified by intrinsic subtype, luminal tumors showed positive AA, while tumors typically of more aggressive nature (HER2+ and basal-like) demonstrated negative AA. Of note, this similar trend was previously reported in studies by Castle et al. [4] and Horvath [5]. Our observation of positive correlation between DNAm AA and expression levels of *ESR1* and *PGR* in the accompanied RNASeq data further supports the hypothesis that an activation of ER signaling pathways, which are key oncogenic drivers of luminal breast cancers, may synergize with age-related epigenetic processes and promote tissue aging through regulating cell proliferation. This is consistent with the concept of ‘breast tissue age’ developed by Malcolm Pike [17], suggesting that lifetime estrogen exposure may drive epigenetic breast aging. Our findings that early age at menarche and high BMI, which are related to cumulative exposure to estrogen, were associated with increased DNAm AA support this hypothesis. Similar associations between epigenetic age and estrogen related exposures were also reported in a previous study by Sehl et al. based on healthy breast tissues [3]. On the other hand, the lower DNAm AA associated with non-luminal tumors is intriguing, given that the number of mutations and SCNAs generally increase with age [18] and non-luminal tumors tend to have higher mutational and SCNA burden than luminal tumors. To better understand subtype-specific differences in epigenetic aging, we further evaluated DNAm age in context of several genomic features and additional aging markers. Consistent with the lower DNAm AA observed among non-luminal cases, we found that lower DNAm AA was also associated with higher tumor mutational burden, *TP53* mutations and pathway function, percent of genome affected by SCNAs, and homologous repair deficiency, all of which are indicators of higher genomic instability. Similar associations of DNAm AA with breast cancer subtypes and *TP53* mutations were previously reported in studies based primarily on TCGA data, where *TP53* mutations were associated with significantly lower age acceleration in five different cancer types including breast cancer [5, 19]. It is unlikely that these associations were entirely driven by the measurement artifact since the associations remained significant when we restricted our analysis to tumors without extensive SCNAs in genomic regions containing the Horvath clock sites. Rather, the more plausible explanation is that extensive genomic alterations especially when coupled with *TP53* inactivation that occur more often in more aggressive subtypes like basal-like tumors may stimulate multiple molecular processes such as cell senescence, chronic inflammation, and epithelial-to-mesenchymal transition, which may in turn disrupt normal age-related processes. For example, although transient cell senescence, which is indicative of functional p53 signaling in response to many forms of DNA damage, is recognized as a tumor suppressor mechanism, persistent senescence accompanied by p53 inactivation would promote a senescence-associated secretory phenotype (SASP), which stimulates the secretion of numerous proinflammatory cytokines and growth factors that may modulate the epigenome and disrupt normal tissue structure and function [20]. In our study, we indeed observed a significantly higher expression of *CDKN2A*, a cell senescence marker, in basal-like tumors as compared to tumors of other subtypes, a finding that we replicated in TCGA breast (Additional file 3: Figure S3) and was reported by Cheng et al. using TCGA data [21]. In addition, oncogene-induced senescent cells could also re-enter the cell cycle and present with a much higher tumor initiation potential (enhanced cancer-cell stemness), resulting in a highly aggressive tumor phenotype [22], such as basal-like tumors. It was shown that stemlike cells had younger epigenetic age [5] and basal-like tumors are known to contain a higher percentage of breast cancer stem cells than the other subtypes [23].

The distinct epigenetic aging patterns observed in basal-like tumors further highlight the biological uniqueness of this subtype and suggest that epigenetic age may be a useful measurement to better understand this subtype. For example, in our analysis, none of the basal-like tumors showed positive DNAm AA. The variations of DNAm AA by cancer type and subtype may also lead to inconsistent results using epigenetic aging in prognosis prediction. Although advanced epigenetic age is often associated with higher risks of disease and mortality in normal and cancerous tissues, lower epigenetic age has been associated with poor survival in breast cancer [19] and glioma [24].

One of the major strengths of our study is the extensive genomic data from breast cancer patients in an East Asian population, which allowed us to comprehensively evaluate epigenetic aging in relation to breast cancer driver mutations, functional pathways (such as estrogen), and other aging markers. In addition, we also investigated the effect of key breast cancer risk factors on epigenetic age, which was rarely examined in previous studies. The major limitation of our study is the small number of subjects, which results in limited statistical power, especially for subtype-stratified analyses. However, we validated all major findings in TCGA (Additional file 3: Figure S3). Another limitation is lack of outcome information, which limited our ability to understand the clinical relevance of our findings.

In conclusion, we conducted a comprehensive evaluation of DNAm AA in relation to breast cancer subtypes, somatic alterations, and major etiologic factors using data derived from an East Asian dataset. Our findings demonstrate similar patterns of Horvath clock in estimating breast tissue age in a non-European population to what were reported in European populations. We replicated the previously reported diverging patterns of DNA methylation-based age acceleration by tumor subtype that were primarily based on European patients, with luminal tumors displaying increased age acceleration while HER2-enriched/basal-like tumors exhibiting age deceleration. By leveraging the extensive genomic data from RNASeq and WGS, we further showed that the different directions in DNAm AA by breast cancer subtypes are largely driven by opposing mechanisms related to estrogen exposures and genomic alterations. Our findings highlight the complexity of epigenetic aging in cancer tissue and the need for additional mechanistic studies.

## Supplementary Information

**Additional file 1** Distribution of DNAm age acceleration by RNA-based functional TP53 mutation status.

**Additional file 2**. Distributions of DNAm age acceleration by genomic features and when stratified by tumor subtype.

**Additional file 3**. DNAm age acceleration in The Cancer Genome Atlas (TCGA) dataset.

**Additional file 4**. Distributions of DNAm age acceleration by genomic features after the exclusion of tumors displaying extensive somatic copy number alterations in the Horvath clocks sites.

**Additional file 5**. Associations between DNAm age acceleration in tumor tissue and tumor features in Hong Kong breast cancer women.

**Additional file 6**. Associations between DNAm age acceleration in tumor tissue and tumor features in The Cancer Genome Atlas breast cancer women.

**Additional file 7.** Breast cancer risk factors in Hong Kong (HKBC) women by DNA methylation (DNAm) age and its acceleration (AA) in normal tissue.

## Data Availability

Sequencing and microarray data generated in the Hong Kong breast cancer study has been deposited in the dbGaP database under Accession Code phs001870.v1.p1. at https://www.ncbi.nlm.nih.gov/projects/gap/cgi-bin/study.cgi?study_id=phs001870.v1.p1.
