# Supplementary material for: DNA methylation age in paired tumor and adjacent normal breast tissue in Chinese women with breast cancer
Source: Clin Epigenetics. 2023 Mar 30;15:55. doi: 10.1186/s13148-023-01465-1 (PMC10062015; doi:10.1186/s13148-023-01465-1)

**Figure S1: Distribution of DNAm age acceleration by RNA-based functional *TP53* mutation status.** Kruskal-Wallis test was used to formally assess median differences by mutation status.


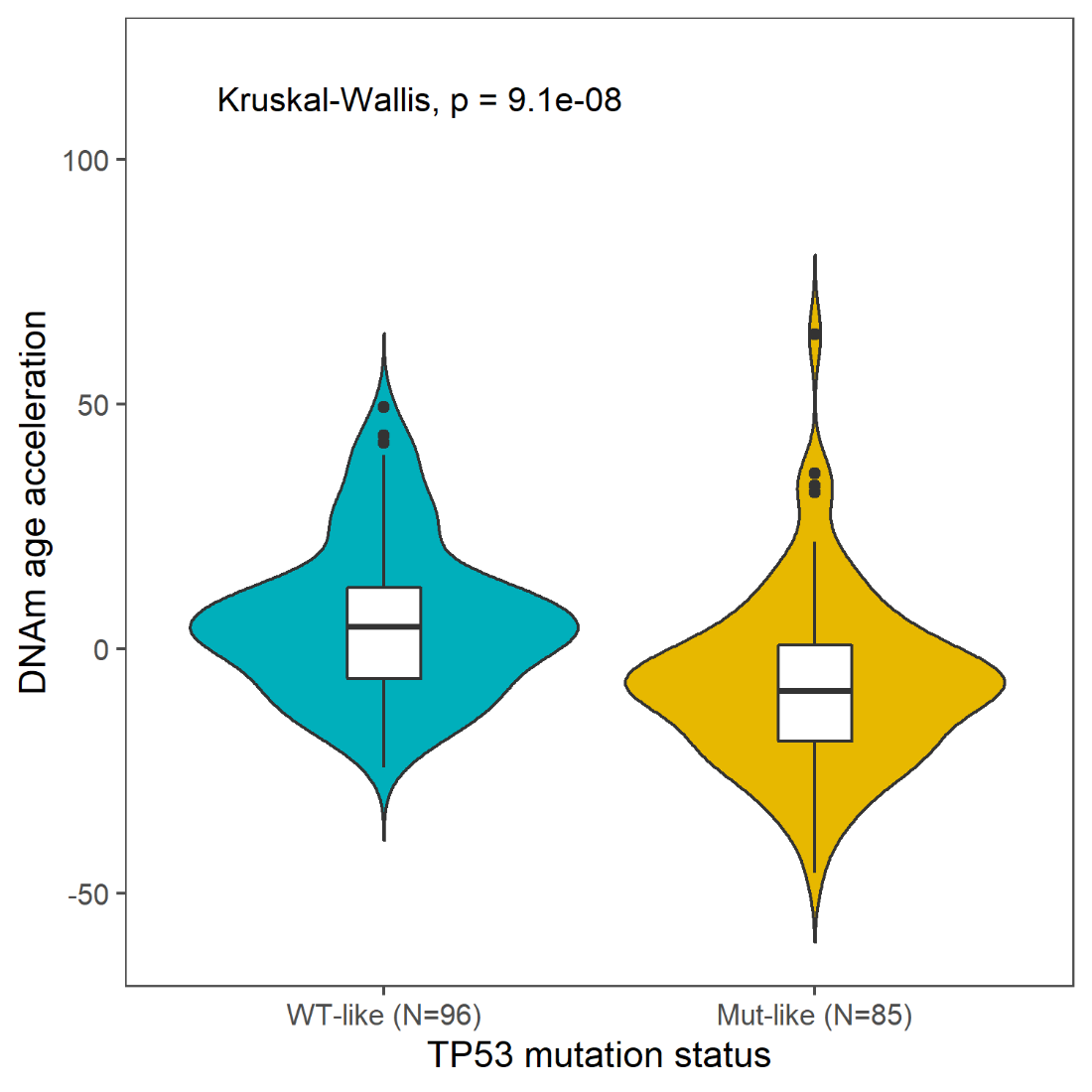

Supplement: Supplementary file 1 — Additional file 1 Distribution of DNAm age acceleration by RNA-based functional TP53 mutation status. [file 13148_2023_1465_MOESM1_ESM.docx]
