# Supplementary material for: DNA methylation age in paired tumor and adjacent normal breast tissue in Chinese women with breast cancer
Source: Clin Epigenetics. 2023 Mar 30;15:55. doi: 10.1186/s13148-023-01465-1 (PMC10062015; doi:10.1186/s13148-023-01465-1)

**Figure S2: Distribution of DNAm age acceleration by genomic features and when stratified by tumor subtype.** A) Distribution of DNAm age acceleration by *ESR1* and *PGR* gene expression; B) Distribution of DNAm age acceleration by DNA-based *TP53* mutation status; C) Distribution of DNAm age acceleration by tumor mutation burden (tumor mutation burden was dichotomized using cut-off point of 1.3); C) Distribution of DNAm age acceleration by HRDetect score; E) Distribution of DNAm age acceleration by percent genome with somatic copy number of alterations (PGS); F) Distribution of DNAm age acceleration by *CDKN2A* gene expression. Kruskal Wallis tests were used to formally assess median differences by a feature. Pearson correlation test was utilized to test for significance of correlation between percent genome with PGS and CDKN2A expression and DNAm age acceleration.

**A**


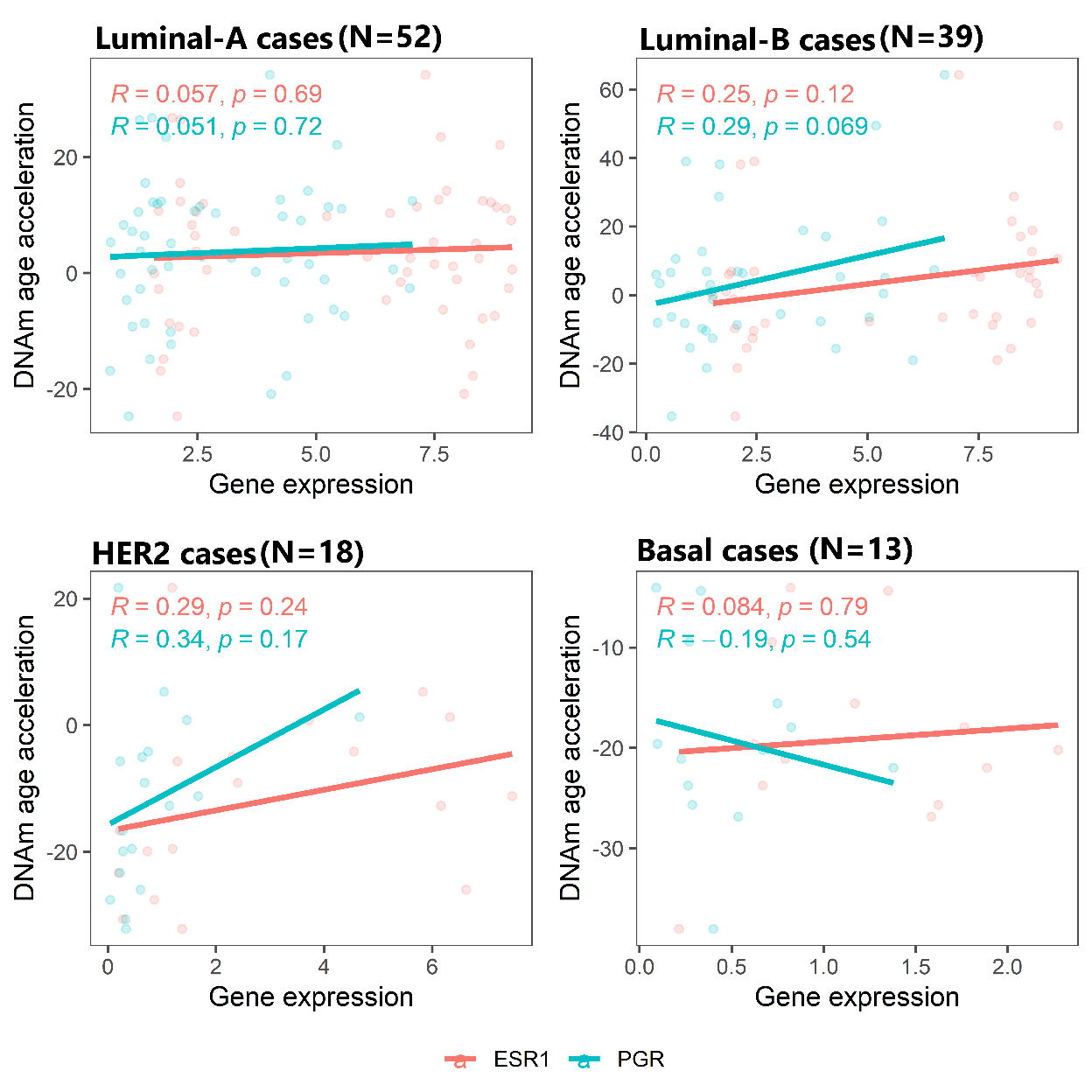


**B**


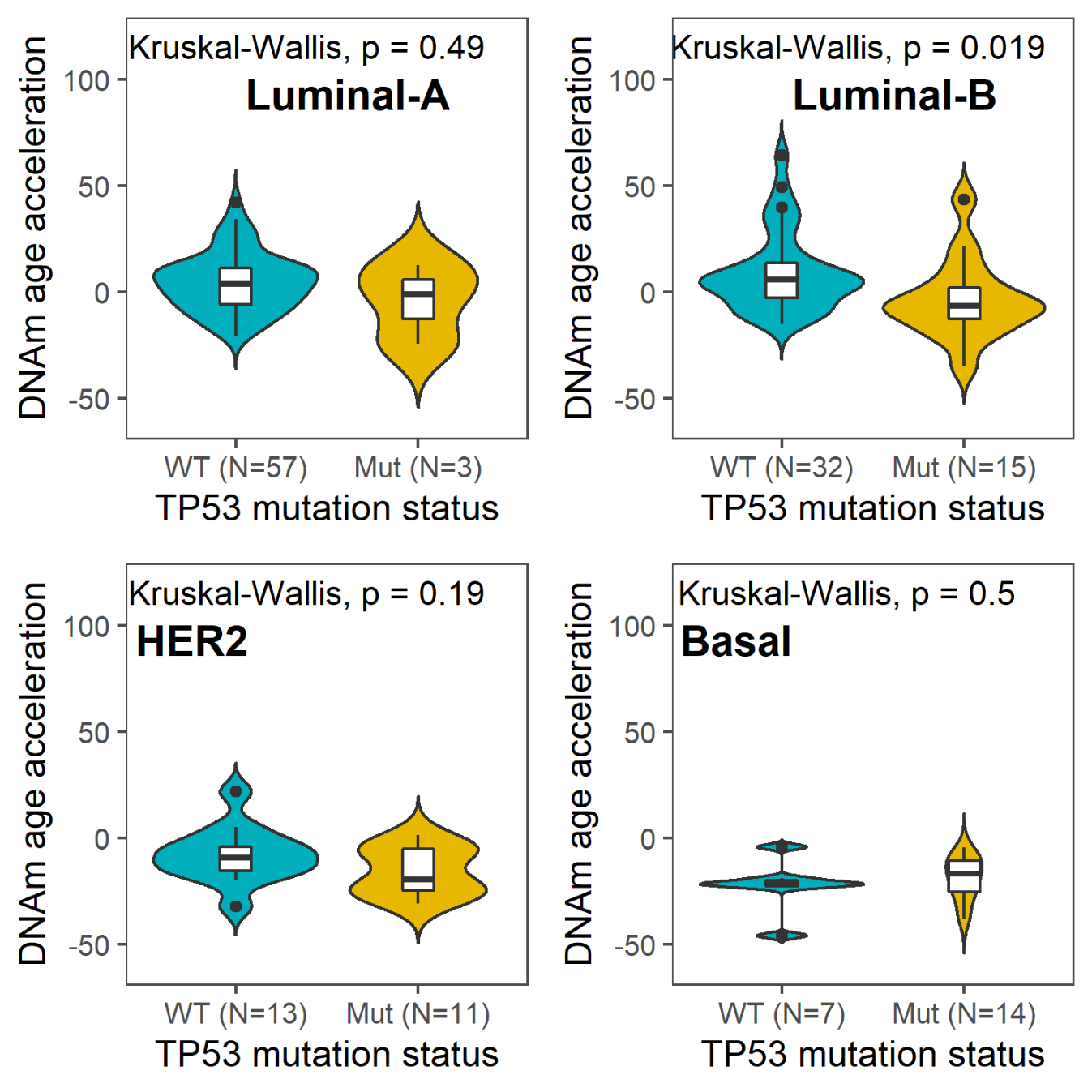


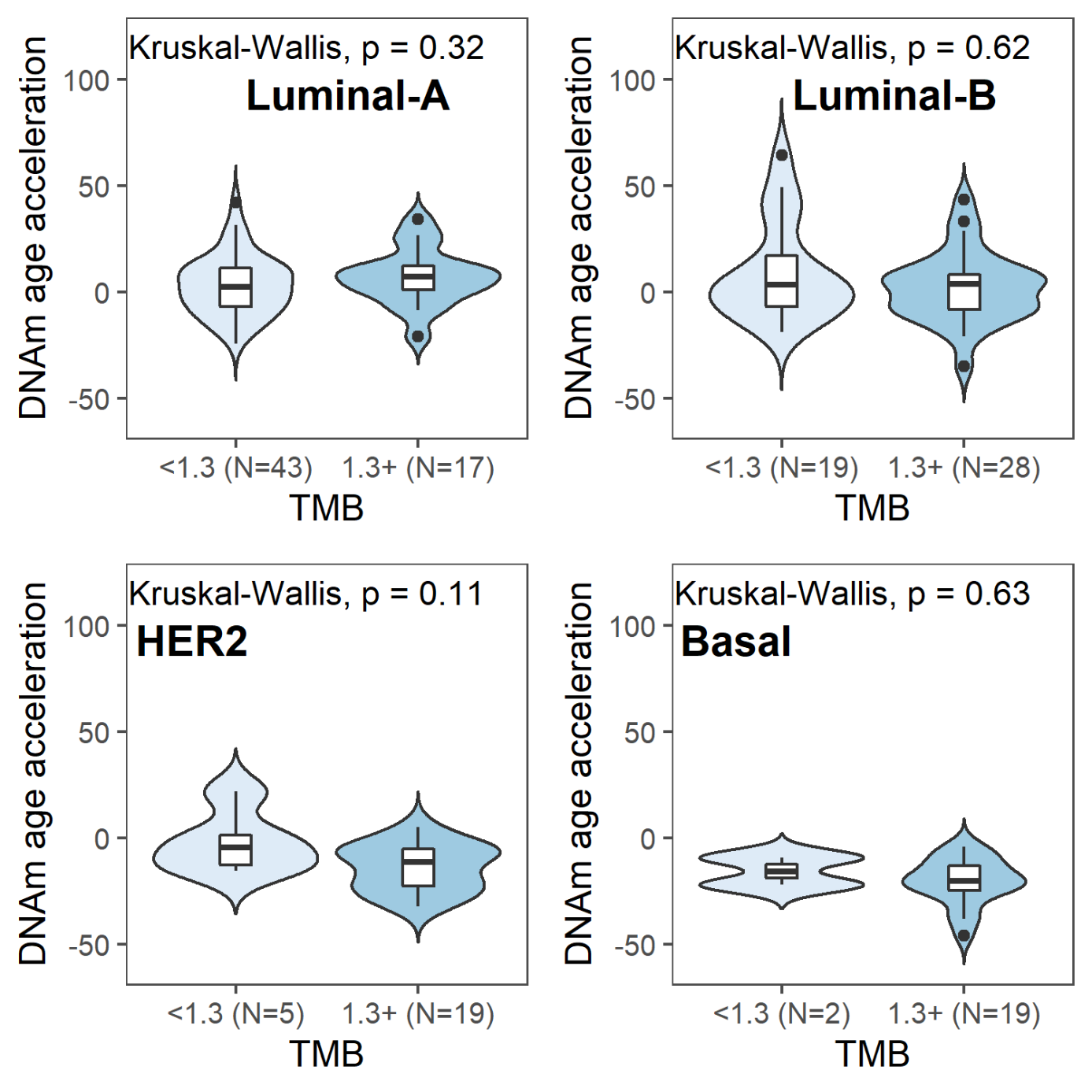


**C**


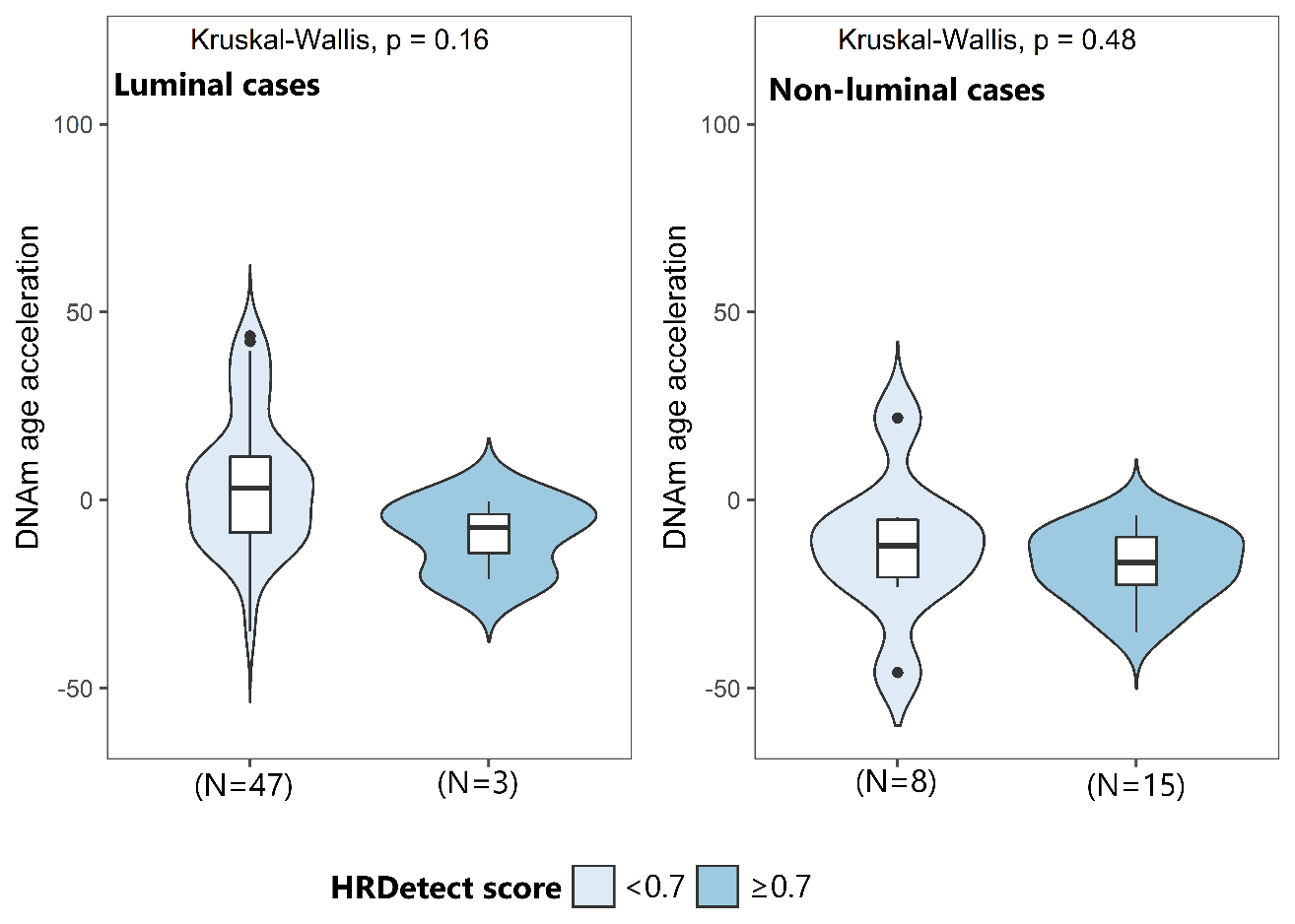


**D**


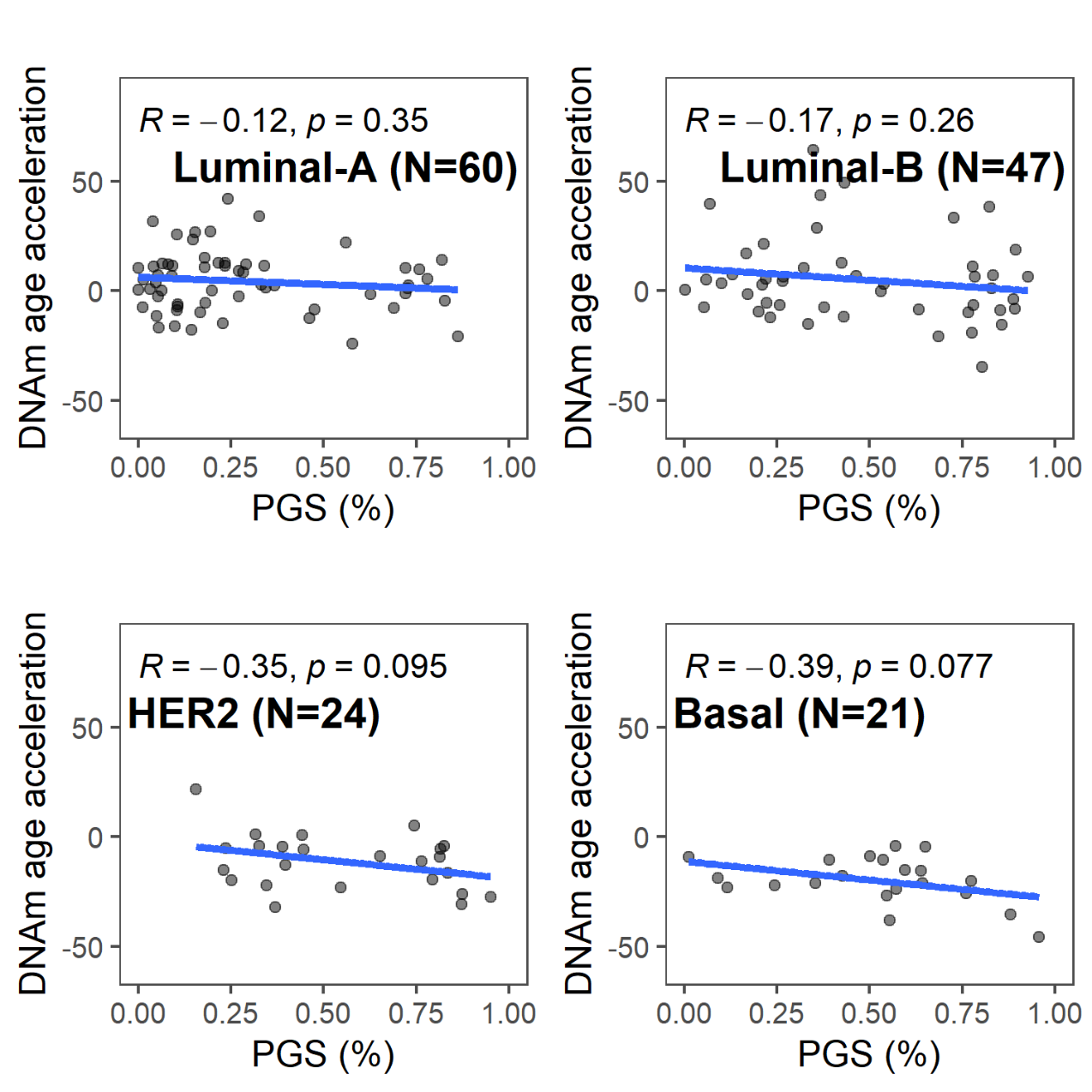


**E**

**F**


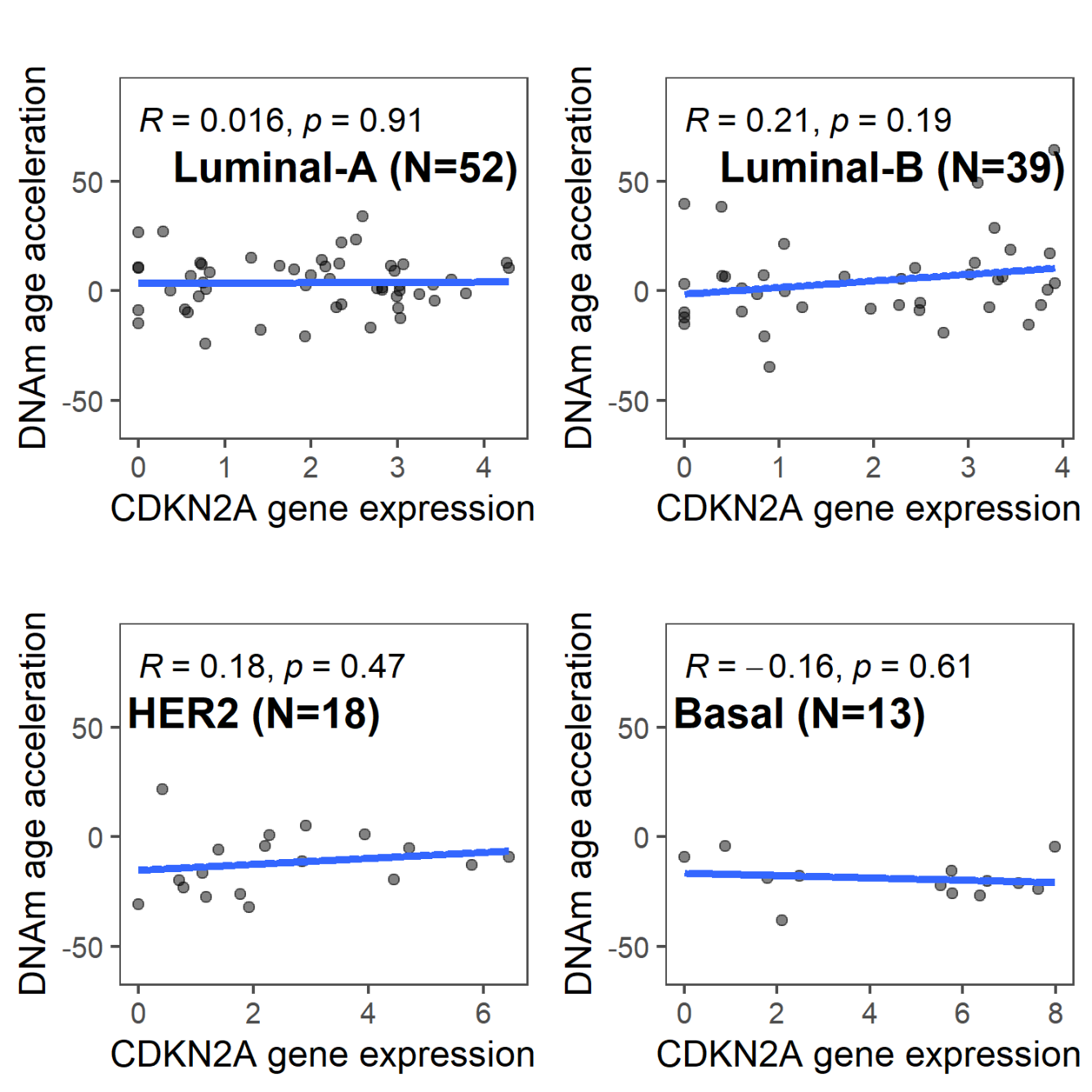

Supplement: Supplementary file 2 — Additional file 2. Distributions of DNAm age acceleration by genomic features and when stratified by tumor subtype. [file 13148_2023_1465_MOESM2_ESM.docx]
