# Supplementary material for: DNA methylation age in paired tumor and adjacent normal breast tissue in Chinese women with breast cancer
Source: Clin Epigenetics. 2023 Mar 30;15:55. doi: 10.1186/s13148-023-01465-1 (PMC10062015; doi:10.1186/s13148-023-01465-1)
Supplement: Supplementary file 3 — Additional file 3. DNAm age acceleration in The Cancer Genome Atlas (TCGA) dataset. [file 13148_2023_1465_MOESM3_ESM.docx]

**Figure S3: DNAm age acceleration in The Cancer Genome Atlas (TCGA) dataset**.

The TCGA dataset utilized in this work has methylation data from breast cancer tumors (n=559) and adjacent normal (n=90) in patients of European ancestry from The Cancer Genome Atlas (TCGA) project. RNASeq data (tumor: n=559 and adjacent normal: n=83) was also available for most of the patients from TCGA. Clinical characteristics were ascertained from medical records for most of the TCGA women. In this replication set, the Infinium HumanMethylation450 BeadChip (Methy450K) array was utilized. DNAm age and its AA were calculated similarly to that in the HKBC study, and the additional genomic analyses in TCGA samples were also run similarly as in HKBC.

A) Distribution of DNAm age acceleration by tissue type; B) Distribution of DNAm age acceleration in tumor tissue by breast cancer subtypes; Associations between DNAm age acceleration and *ESR1* and *PGR* gene expression in C) normal and D) tumor tissue; Distribution of DNAm age acceleration by E) DNA-based *TP53* mutation status and F) RNA-based functional *TP53* mutation status; G) Distribution of DNAm age acceleration by tumor mutation burden categorized using tertials; H) Distribution of *CDKN2A* gene expression in normal and tumor tissue, when stratified by tumor subtype.

**B**

**A**


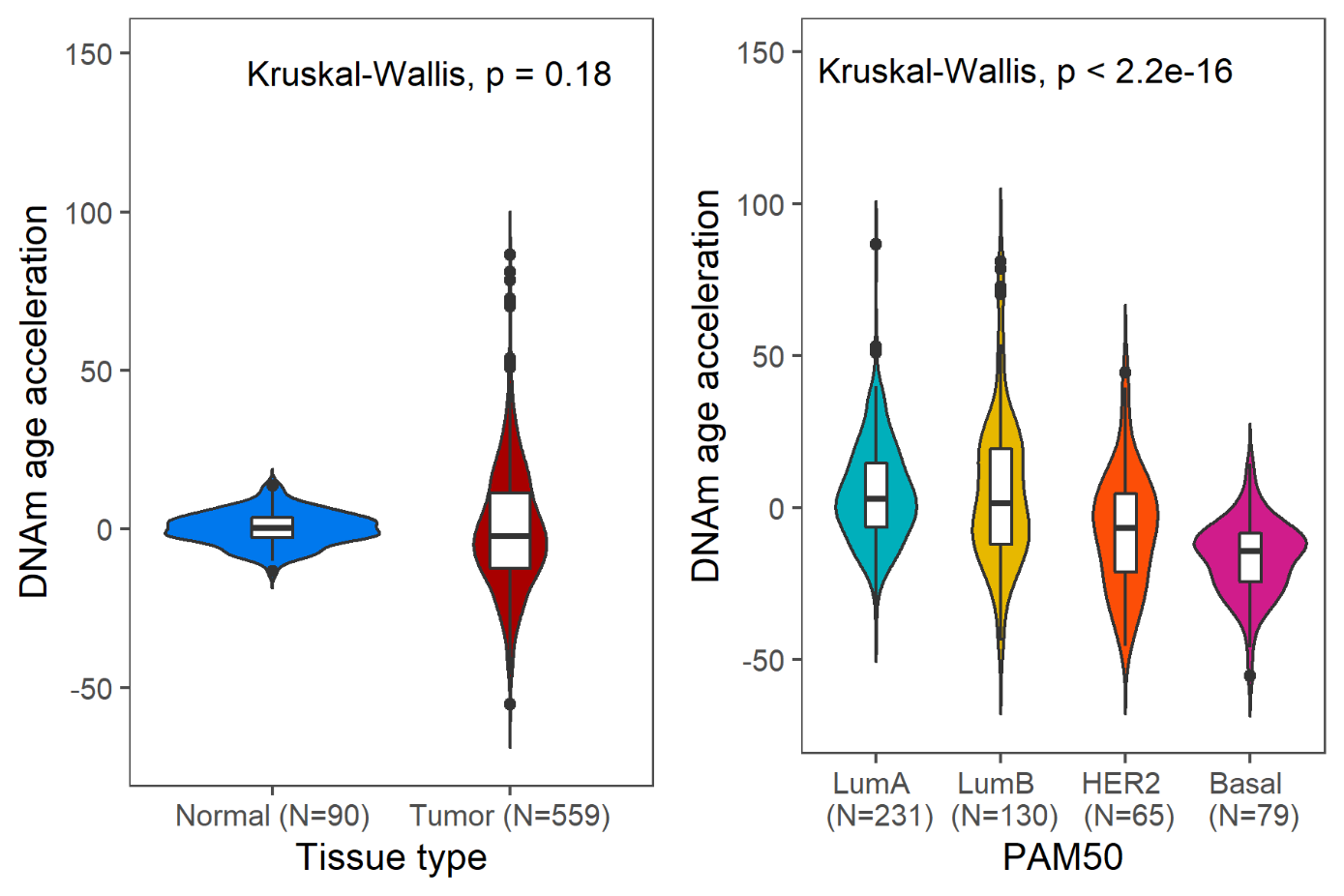


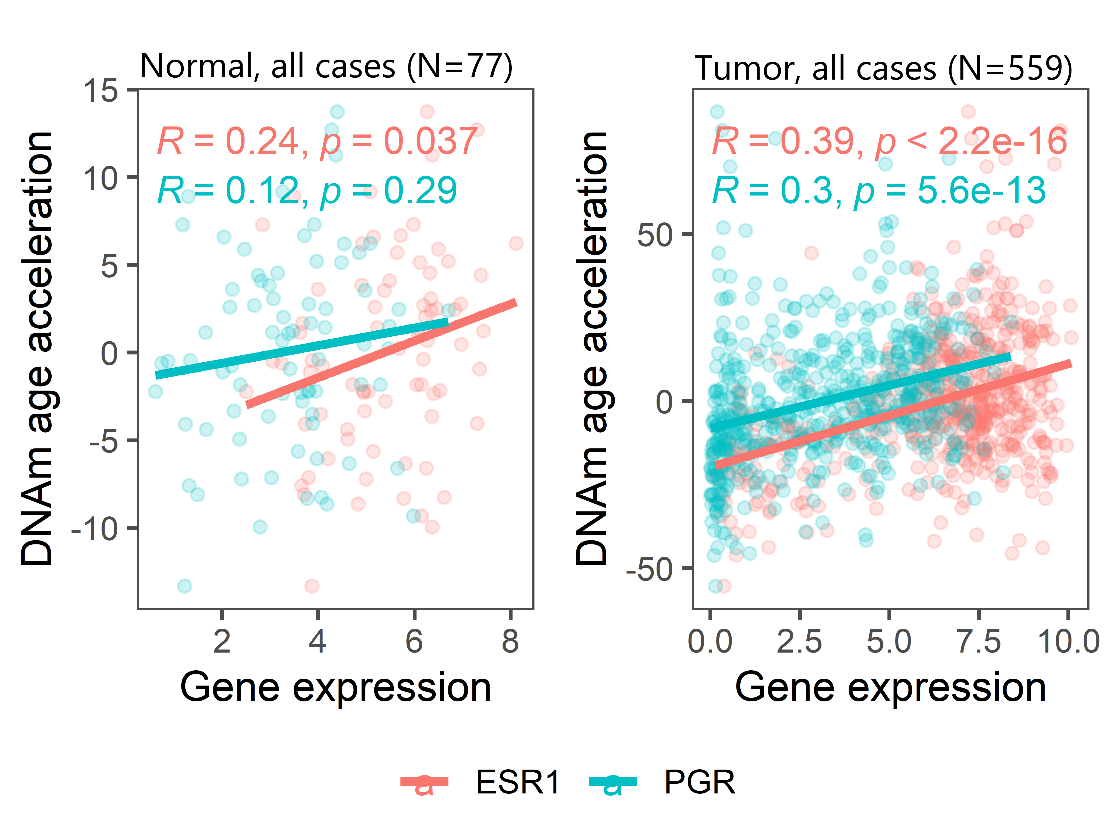


**D**

**C**

**F**

**E**

**
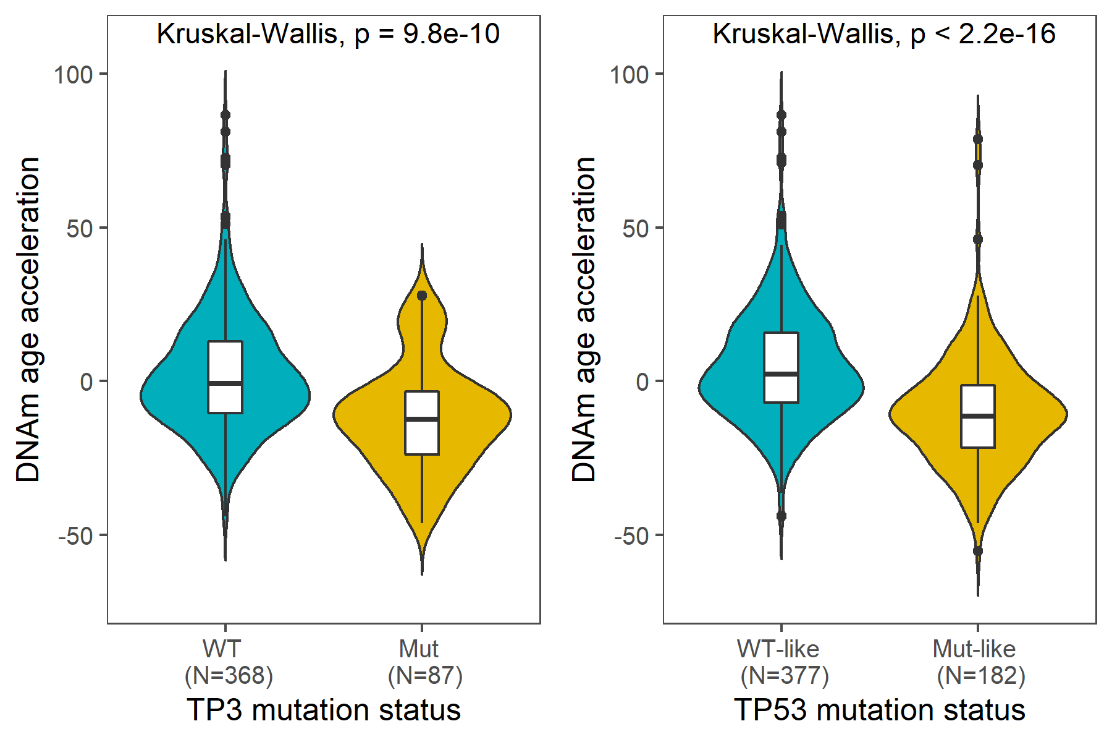
**

**G**

**
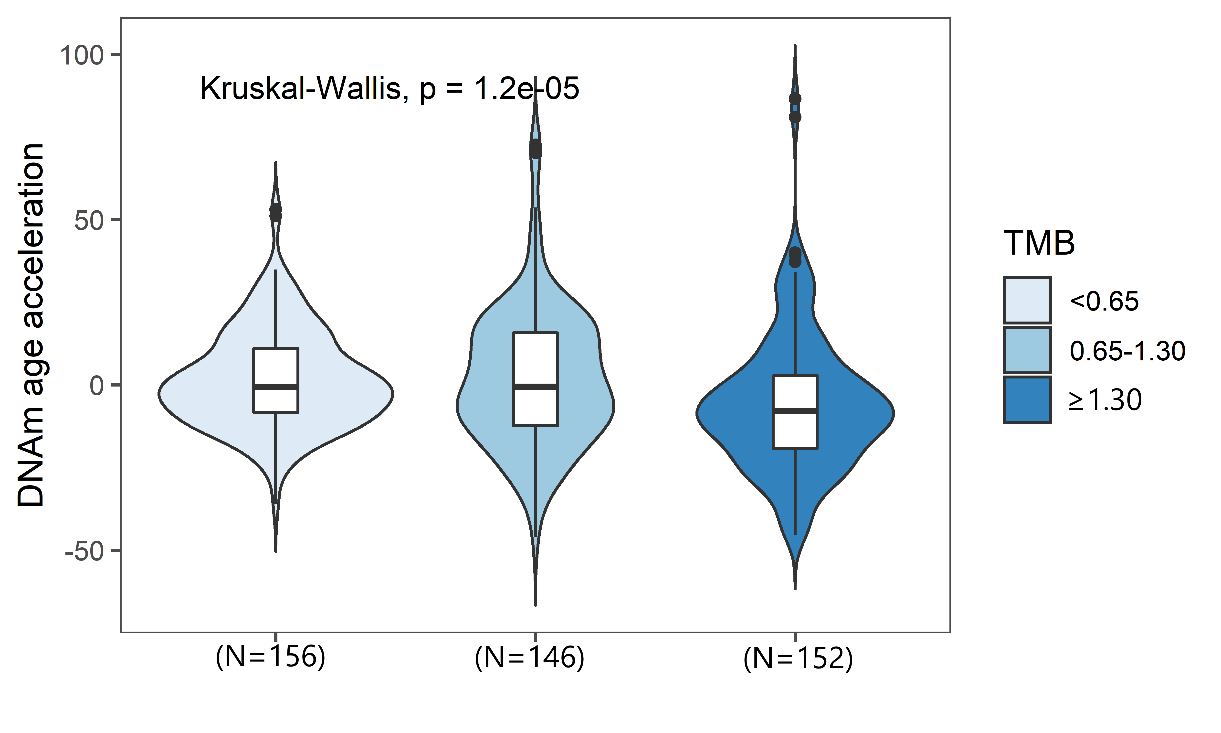
**

**H**

**
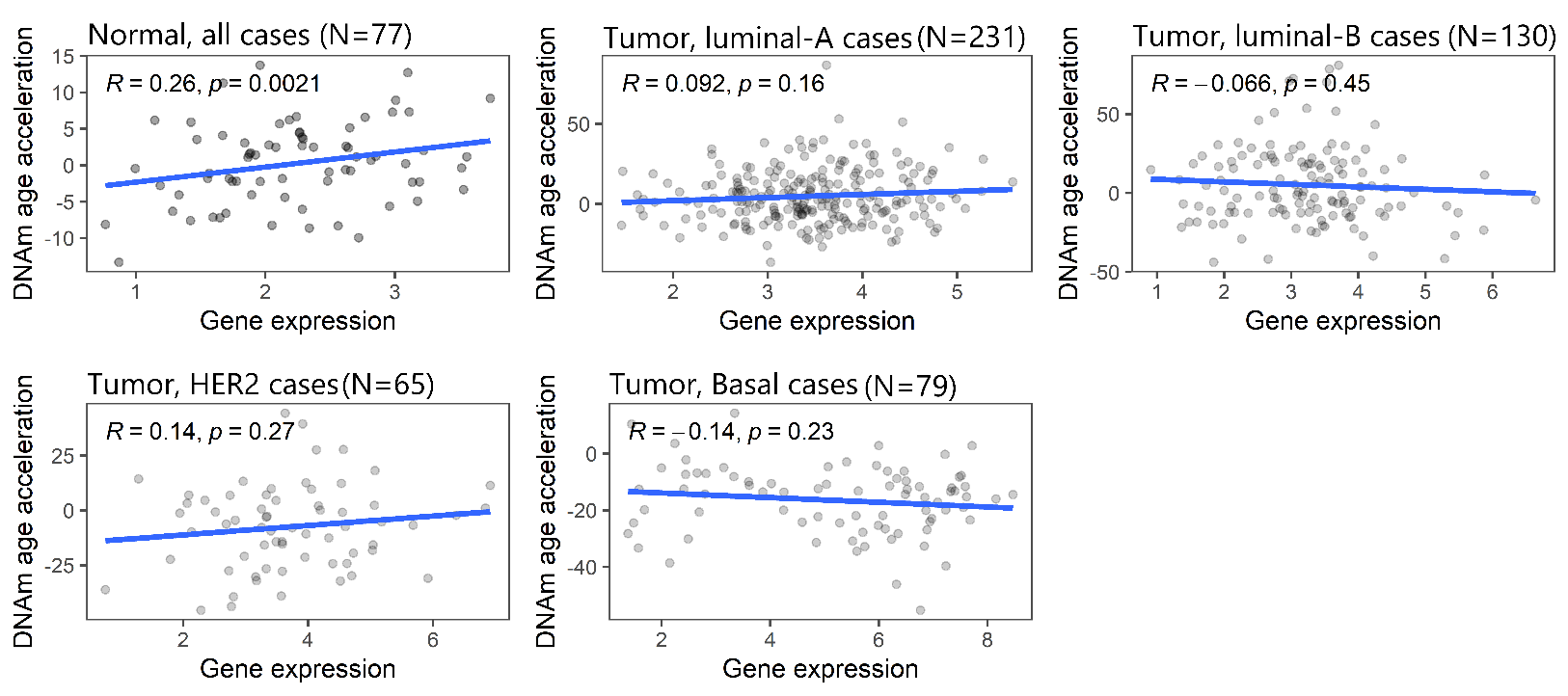
**

**
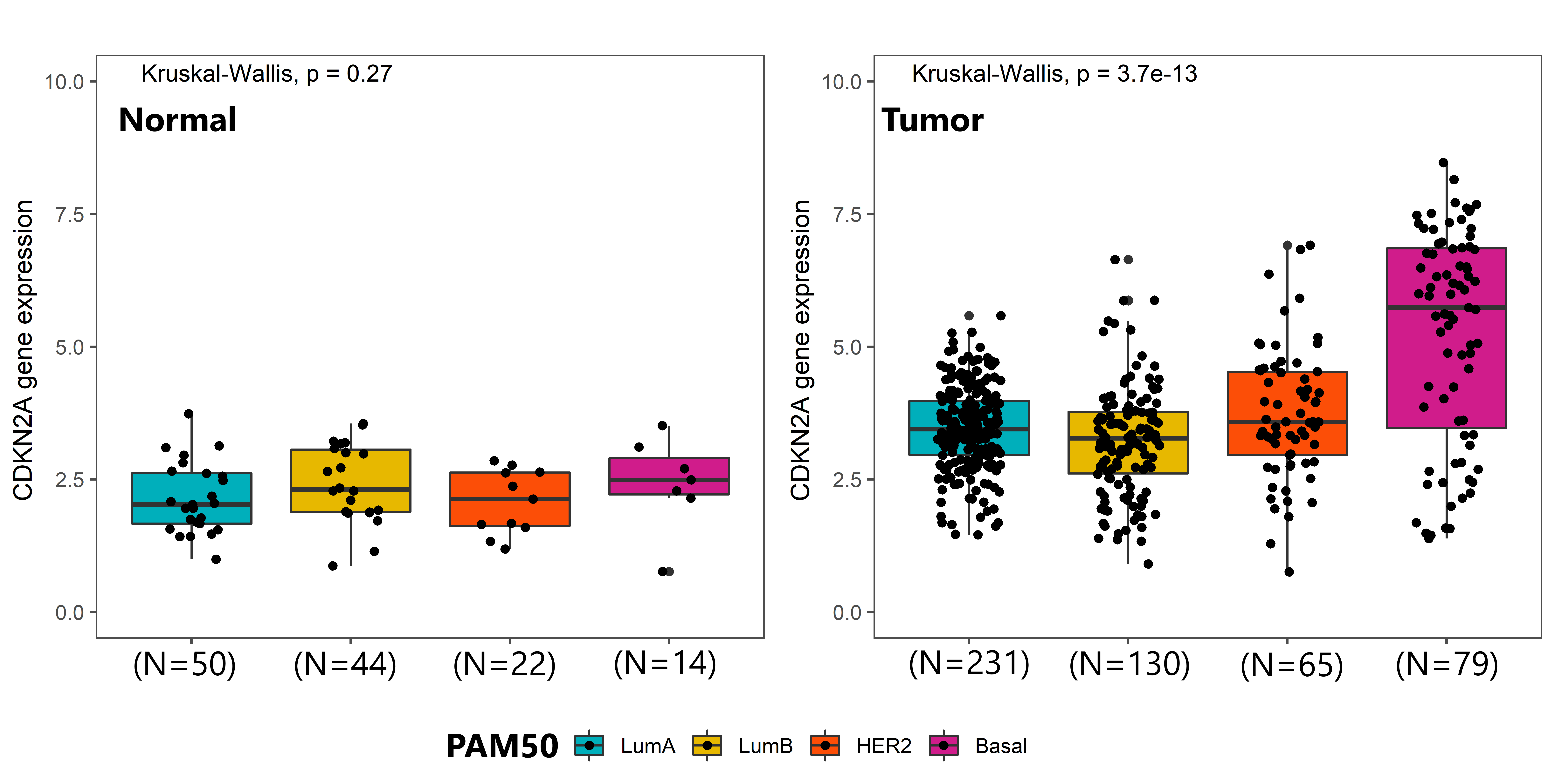
**

**I\I**
