# Supplementary material for: DNA methylation age in paired tumor and adjacent normal breast tissue in Chinese women with breast cancer
Source: Clin Epigenetics. 2023 Mar 30;15:55. doi: 10.1186/s13148-023-01465-1 (PMC10062015; doi:10.1186/s13148-023-01465-1)
Supplement: Supplementary file 4 — Additional file 4. Distributions of DNAm age acceleration by genomic features after the exclusion of tumors displaying extensive somatic copy number alterations in the Horvath clocks sites. [file 13148_2023_1465_MOESM4_ESM.docx]

**Figure S4: Distributions of DNAm age acceleration by genomic features after the exclusion of tumors displaying extensive somatic copy number alterations in the Horvath clocks sites.** A) Distribution of DNAm age acceleration by PAM50 tumor subtype; B) Distribution of DNAm age acceleration by DNA-based *TP53* mutation status. Kruskal-Wallis tests were used to formally assess median differences by each feature separately.


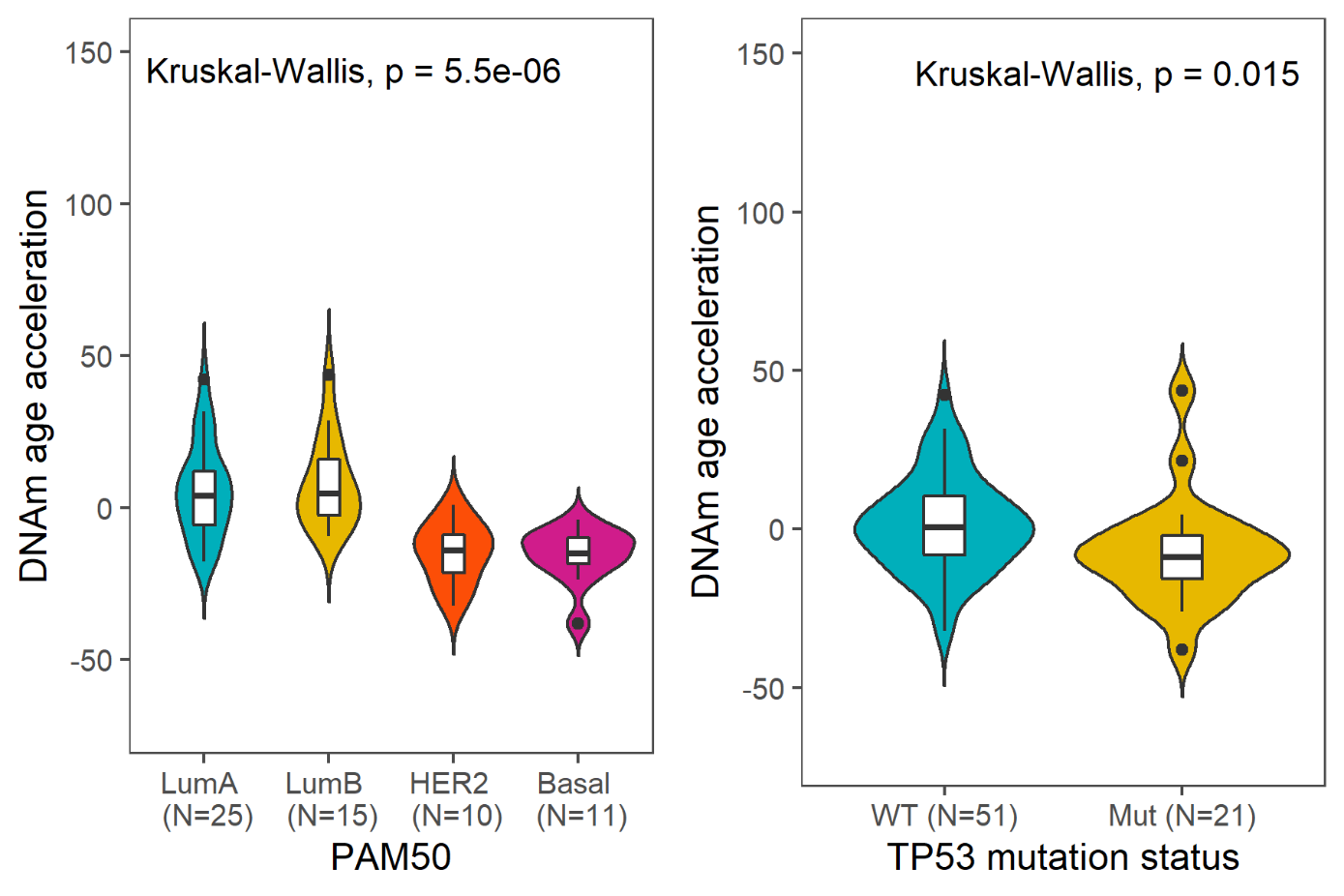


**B**

**A**
