# Supplementary material for: DNA methylation age in paired tumor and adjacent normal breast tissue in Chinese women with breast cancer
Source: Clin Epigenetics. 2023 Mar 30;15:55. doi: 10.1186/s13148-023-01465-1 (PMC10062015; doi:10.1186/s13148-023-01465-1)
Supplement: Supplementary file 5 — Additional file 5. Associations between DNAm age acceleration in tumor tissue and tumor features in Hong Kong breast cancer women. [file 13148_2023_1465_MOESM5_ESM.docx]

**Table S1: Associations between DNAm age acceleration in tumor tissue and tumor characteristics in Hong Kong breast cancer women.**

| Characteristic | β (SE) | P-value^a^ |
| --- | --- | --- |
| Among luminal-A cases (N= 52) |  |  |
| *TP53*- Mut vs. *TP53*- WT | -6.15 (7.5) | 0.414 |
| Tumor mutation burden | 3.23 (2.2) | 0.154 |
| PGS^b^ | -11.25 (7.5) | 0.140 |
| *ESR1* | 0.27 (0.6) | 0.659 |
| Among luminal-B cases (N= 39) |  |  |
| *TP53*- Mut vs. *TP53*- WT | -16.61 (7.1) | **0.026** |
| Tumor mutation burden | -0.48 (0.3) | 0.154 |
| PGS^b^ | 0.58 (10.3) | 0.955 |
| *ESR1* | 1.27 (1.0) | 0.192 |
| Among HER2 cases (N= 18) |  |  |
| *TP53*- Mut vs. *TP53*- WT | -5.32 (6.4) | 0.420 |
| Tumor mutation burden | -3.18 (3.4) | 0.369 |
| PGS^b^ | -15.58 (14.4) | 0.300 |
| *ESR1* | 1.10 (1.2) | 0.389 |
| Among basal cases (N= 13) |  |  |
| *TP53*- Mut vs. *TP53*- WT | -0.40 (5.3) | 0.942 |
| Tumor mutation burden | -1.64 (0.6) | **0.032** |
| PGS^b^ | 6.94 (11.8) | 0.573 |
| *ESR1* | -6.08 (5.2) | 0.277 |

aResults were obtained from running multivariate linear regression analysis, where age acceleration was treated as the outcome.

^b^Percent genome with copy number alterations.
