# Supplementary material for: DNA methylation age in paired tumor and adjacent normal breast tissue in Chinese women with breast cancer
Source: Clin Epigenetics. 2023 Mar 30;15:55. doi: 10.1186/s13148-023-01465-1 (PMC10062015; doi:10.1186/s13148-023-01465-1)
Supplement: Supplementary file 6 — Additional file 6. Associations between DNAm age acceleration in tumor tissue and tumor features in The Cancer Genome Atlas breast cancer women. [file 13148_2023_1465_MOESM6_ESM.docx]

**Table S2: Associations between DNAm age acceleration in tumor tissue and tumor features in The Cancer Genome Atlas breast cancer women.**

| Feature | β (SE) | P-value^a^ |
| --- | --- | --- |
| Among all cases (N= 454) |  |  |
| *TP53*- Mut vs. *TP53*- WT | -7.72 (2.2) | **0.0005** |
| *ESR1* | 2.58 (0.4) | **<.0001** |
| Tumor mutation burden | -0.31 (0.2) | 0.134 |
| Among luminal-A cases (N= 187) |  |  |
| *TP53*- Mut vs. *TP53*- WT | 4.30 (5.9) | 0.469 |
| *ESR1* | 0.21 (1.1) | 0.854 |
| Tumor mutation burden | -0.13 (0.3) | 0.659 |
| Among luminal-B cases (N= 110) |  |  |
| *TP53*- Mut vs. *TP53*- WT | -14.11 (6.4) | **0.030** |
| *ESR1* | 1.23 (2.3) | 0.588 |
| Tumor mutation burden | -0.32 (0.7) | 0.641 |
| Among HER2 cases (N= 55) |  |  |
| *TP53*- Mut vs. *TP53*- WT | -12.83 (4.4) | **0.005** |
| *ESR1* | 2.50 (1.0) | **0.018** |
| Tumor mutation burden | -1.15 (0.5) | **0.025** |
| Among basal cases (N= 65) |  |  |
| *TP53*- Mut vs. *TP53*- WT | 0.29 (2.8) | 0.916 |
| *ESR1* | 0.43 (1.3) | 0.744 |
| Tumor mutation burden | -0.01 (0.3) | 0.970 |
