# Supplementary material for: DNA methylation age in paired tumor and adjacent normal breast tissue in Chinese women with breast cancer
Source: Clin Epigenetics. 2023 Mar 30;15:55. doi: 10.1186/s13148-023-01465-1 (PMC10062015; doi:10.1186/s13148-023-01465-1)
Supplement: Supplementary file 7 — Additional file 7. Breast cancer risk factors in Hong Kong (HKBC) women by DNA methylation (DNAm) age and its acceleration (AA) in normal tissue. [file 13148_2023_1465_MOESM7_ESM.docx]

**Table S3: Breast cancer risk factors in Hong Kong (HKBC) women by DNA methylation (DNAm) age and its acceleration (AA) in normal tissue.**

|  |  | **DNAm age** |  | **DNAm AA** |  |
| --- | --- | --- | --- | --- | --- |
| Characteristic | **N** | **Mean (SD)** | **P-value^a^** | **Mean (SD)** | **P-value^a^** |
| Age, year |  |  |  |  |  |
| <50 | 36 | 59.3 (5.1) | **<.00001** | 0.10 (3.4) | 0.339 |
| 50-60 | 53 | 65.1 (4.8) |  | 0.50 (4.4) |  |
| ≥60 | 99 | 71.0 (6.0) |  | -0.31 (4.5) |  |
| BMI^b^, kg/m^2^ |  |  |  |  |  |
| <25 | 92 | 66.1 (7.3) | 0.087 | -0.29 (4.7) | 0.114 |
| 25-30 | 50 | 68.6 (6.0) |  | 0.99 (3.8) |  |
| ≥30 | 22 | 65.7 (7.8) |  | 0.66 (2.6) |  |
| Age at menarche, year |  |  |  |  |  |
| <14 | 97 | 66.6 (6.6) | 0.890 | 0.43 (3.8) | **0.035** |
| ≥14 | 80 | 67.3 (6.9) |  | -0.45 (4.4) |  |
| Parity |  |  |  |  |  |
| Nulliparous | 16 | 65.3 (9.3) | 0.664 | 1.12 (3.7) | 0.128 |
| Parous | 163 | 67.4 (6.9) |  | -0.05 (4.4) |  |
| Age at first birth^c^, year |  |  |  |  |  |
| <25 | 60 | 68.2 (7.7) | 0.187 | 0.17 (4.3) | 0.792 |
| ≥25 | 92 | 66.9 (6.6) |  | 0.06 (4.4) |  |
| Menopausal status |  |  |  |  |  |
| Pre | 41 | 60.6 (4.5) | **<.0001** | -0.03 (3.4) | 0.903 |
| Post | 136 | 69.4 (6.0) |  | 0.12 (4.5) |  |
| Age at menopause^d^, year |  |  |  |  |  |
| ≤50 | 62 | 68.7 (6.3) | 0.359 | -0.28 (4.5) | 0.926 |
| >50 | 51 | 69.5 (4.2) |  | -0.42 (3.7) |  |
| ^a^Result from Wilcoxon or Kruskal-Wallis test | | | | |  |
| ^b^BMI= Body mass index | | | | |  |
| ^c^Among parous women  ^d^Among post-menopausal women | | | | |  |
